# Ensilication preserves high-molecular weight native DNA for clinical long-read sequencing

**DOI:** 10.1186/s13059-026-04137-4

**Published:** 2026-06-15

**Authors:** Alexis Ferrasse, Rodrigo Mendez, John E. Gorzynski, Chloe Reuter, Jennefer N. Carter, Michael Blas, Undiagnosed Diseases Network, John E. Gorzynski, Chloe Reuter, Alyssa A. Tran, Arjun Tarakad, Ashok Balasubramanyam, Brendan H. Lee, Carlos A. Bacino, Daryl A. Scott, Elaine Seto, Gary D. Clark, Hongzheng Dai, Hsiao-Tuan Chao, Ivan Chinn, James P. Orengo, Jill A. Rosenfeld, Kim Worley, Lindsay C. Burrage, Lisa T. Emrick, Lorraine Potocki, Monika Weisz Hubshman, Richard A. Lewis, Ronit Marom, Seema R. Lalani, Shamika Ketkar, Tiphanie P. Vogel, William J. Craigen, Jared Sninsky, Lauren Blieden, Sandesh Nagamani, Hugo J. Bellen, Michael F. Wangler, Oguz Kanca, Shinya Yamamoto, Christine M. Eng, Patricia A. Ward, Pengfei Liu, Adeline Vanderver, Cara Skraban, Edward Behrens, Gonench Kilich, Kathleen Sullivan, Kelly Hassey, Ramakrishnan Rajagopalan, Rebecca Ganetzky, Vishnu Cuddapah, Anna Raper, Daniel J. Rader, Giorgio Sirugo, Anne Slavotinek, Christopher Mayhew, Eneida Mendonca, Ziyuan Guo, Allyn McConkie-Rosell, Kelly Schoch, Mohamad Mikati, Nicole M. Walley, Rebecca C. Spillmann, Vandana Shashi, Alan H. Beggs, Calum A. MacRae, David A. Sweetser, Deepak A. Rao, Edwin K. Silverman, Elizabeth L. Fieg, Frances High, Gerard T. Berry, Ingrid A. Holm, J. Carl Pallais, Joan M. Stoler, Joseph Loscalzo, Lance H. Rodan, Laurel A. Cobban, Lauren C. Briere, Matthew Coggins, Melissa Walker, Richard L. Maas, Susan Korrick, Jessica Douglas, Cecilia Esteves, Emily Glanton, Isaac S. Kohane, Kimberly LeBlanc, Shamil R. Sunyaev, Shilpa N. Kobren, Brett H. Graham, Erin Conboy, Francesco Vetrini, Kayla M. Treat, Khurram Liaqat, Lili Mantcheva, Stephanie M. Ware, Kathleen Page, Paul Auwaerter, Yuka Manabe, Carlos A. Pardo-Villamizar, Julie Hoover-Fong, Philip Dane Witmer, Winston Timp, Matthew Robinson, Zackary Dov Berger, Elizabeth Wohler, Nara Sobreira, Arian Nouraee, Carlos Prada, Erica Davis, Kai Lee Yap, Kelly Regan-Fendt, María Paula Silva, Patrick McMullen, Breanna Mitchell, Brendan C. Lanpher, Devin Oglesbee, Eric Klee, Filippo Pinto e Vairo, Ian R. Lanza, Kahlen Darr, Lindsay Mulvihill, Lisa Schimmenti, Queenie Tan, Surendra Dasari, Abdul Elkadri, Brett Bordini, Donald Basel, James Verbsky, Julie McCarrier, Michael Muriello, Michael T. Zimmermann, Adriana Rebelo, Carson A. Smith, Deborah Barbouth, Guney Bademci, Joanna M. Gonzalez, Kumarie Latchman, LéShon Peart, Mustafa Tekin, Nicholas Borja, Stephan Zuchner, Stephanie Bivona, Willa Thorson, Herman Taylor, Rakale C. Quarells, Ayuko Iverson, Bruce Gelb, Charlotte Cunningham-Rundles, Eric Gayle, Joanna Jen, Louise Bier, Mafalda Barbosa, Manisha Balwani, Mariya Shadrina, Rachel Evard, Saskia Shuman, Susan Shin, Vaidehi Jobanputra, Andrea Gropman, Barbara N. Pusey Swerdzewski, Camilo Toro, Colleen E. Wahl, Donna Novacic, Ellen F. Macnamara, John J. Mulvihill, Maria T. Acosta, Precilla D’Souza, Valerie V. Maduro, Ben Afzali, Ben Solomon, Cynthia J. Tifft, David R. Adams, Elizabeth A. Burke, Francis Rossignol, Heidi Wood, Jiayu Fu, Joie Davis, Leoyklang Petcharet, Lynne A. Wolfe, Margaret Delgado, Marie Morimoto, Marla Sabaii, MayChristine V. Malicdan, Neil Hanchard, Orpa Jean-Marie, Wendy Introne, William A. Gahl, Yan Huang, Andrew Stergachis, Danny E. Miller, Elisabeth Rosenthal, Elizabeth Blue, Elsa Balton, Emily Shelkowitz, Eric Allenspach, Fuki M. Hisama, Gail P. Jarvik, Ghayda Mirzaa, Ian Glass, Kathleen A. Leppig, Katrina Dipple, Mark Wener, Martha Horike-Pyne, Michael Bamshad, Peter Byers, Runjun Kumar, Seth Perlman, Sirisak Chanprasert, Virginia Sybert, Wendy Raskind, Nitsuh K. Dargie, Chun-Hung Chan, Francisco Bustos velasq, Isum Ward, Jason Schend, Jennifer Morgan, Megan Bell, Miranda Leitheiser, Mohamad Saifeddine, Paul Berger, Rachel Li, Taylor Beagle, Alexander Miller, Beatriz Anguiano, Beth A. Martin, Brianna Tucker, Devon Bonner, Elijah Kravets, Hector Rodrigo Mendez, Holly K. Tabor, Jacinda B. Sampson, Jason Hom, Jennefer N. Kohler, Jennifer Schymick, Kevin S. Smith, Laura Keehan, Laurens Wiel, Meghan C. Halley, Mia Levanto, Page C. Goddard, Paul G. Fisher, Rachel A. Ungar, Raquel L. Alvarez, Sara Emami, Shruti Marwaha, Stephen B. Montgomery, Suha Bachir, Tanner D. Jensen, Taylor Maurer, Terra R. Coakley, Anna Hurst, Brandon M. Wilk, Bruce Korf, Elizabeth A. Worthey, Kaitlin Callaway, Martin Rodriguez, Pongtawat Lertwilaiwittaya, Reaford Blackburn, Tammi Skelton, Tarun K. K. Mamidi, Teneasha Washington, Andrew B. Crouse, Jordan Whitlock, Mariko Nakano-Okuno, Matthew Might, William E. Byrd, Albert R. La Spada, Changrui Xiao, Elizabeth C. Chao, Eric Vilain, Jose Abdenur, Kirsten Blanco, Maija-Rikka Steenari, Rebekah Barrick, Richard Chang, Sanaz Attaripour, Suzanne Sandmeyer, Tahseen Mozaffar, Alden Huang, Andres Vargas, Bianca E. Russell, Brent L. Fogel, Esteban C. Dell’Angelica, George Carvalho, Julian A. Martínez-Agosto, Layal F. Abi Farraj, Manish J. Butte, Martin G. Martin, Naghmeh Dorrani, Neil H. Parker, Rosario I. Corona, Stanley F. Nelson, Yigit Karasozen, Dana Sayer, Jennifer Tousseau, Aaron Quinlan, Alistair Ward, Ashley Andrews, Corrine K. Welt, Dave Viskochil, Erin E. Baldwin, John Carey, Justin Alvey, Lorenzo Botto, Nicola Longo, Paolo Moretti, Rebecca Overbury, Russell Butterfield, Steven Boyden, Thomas J. Nicholas, Matt Velinder, Gabor Marth, Pinar Bayrak-Toydemir, Rong Mao, Monte Westerfield, John A. Phillips, Kimberly Ezell, Lynette Rives, Rizwan Hamid, Alyson Krokosky, Ashley McMinn, Cathy Shyr, Eric Gamazon, Joy D. Cogan, Lakshitha Perera, Lisa Bastarache, Mary Koziura, Thomas Cassini, Alex Paul, Dana Kiley, Daniel Wegner, Erin McRoy, Jennifer Wambach, Kathy Sisco, Patricia Dickson, F. Sessions Cole, Dustin Baldridge, Jimann Shin, Lilianna Solnica-Krezel, Stephen C. Pak, Timothy Schedl, Allen Bale, Carol Oladele, Caroline Hendry, Emily Wang, Hua Xu, Hui Zhang, Lauren Jeffries, María José Ortuño Romero, Mark Gerstein, Michele Spencer-Manzon, Monkol Lek, Nada Derar, Odelya Kaufman, Shrikant Mane, Teodoro Jerves Serrano, Vasilis Vasiliou, Winston Halstead, Yong-Hui Jiang, Jonathan A. Bernstein, Matthew T. Wheeler, Euan A. Ashley, Jonathan A. Bernstein, Matthew T. Wheeler, James L. Banal, Euan A. Ashley

**Affiliations:** 1 Department of Medicine, Division of Cardiovascular Medicine, Stanford University, Stanford, CA USA; 2 Stanford Center for Undiagnosed Diseases, Stanford, CA USA; 3 Department of Genetics, Stanford University School of Medicine, Stanford, CA USA; 4 Division of Medical Genetics, Department of Pediatrics, Stanford University School of Medicine, Stanford, CA USA; 5 Cache DNA, Inc., San Carlos, CA USA

## Abstract

**Background:**

Native long-read DNA sequencing simultaneously captures genetic variants and epigenetic modifications from single molecules, but preserving molecular length and base modifications currently depends on cold-chain infrastructure that limits access to well-resourced settings.

**Results:**

We demonstrate that ensilication, the encapsulation of DNA within silica matrices, preserves DNA at ambient temperature for 30 days with sequencing performance equivalent to conventional − 80 °C freezing. Across three Genome-in-a-Bottle reference genomes, ensilicated and frozen samples show no significant differences in read length (N50 ~ 8,000–11,000 bp), variant-calling accuracy, or genome-wide CpG methylation. Single-read methylation calls benchmarked against an independent bisulfite-sequencing reference confirm that ensilication introduces no detectable bias, with per-read accuracy differing by less than 0.4% between preservation conditions. Ensilicated DNA tolerates repeated handling better than frozen samples and maintains fragment integrity under accelerated weathering. In two patients with rare genetic disorders, ambient-preserved DNA resolves a de novo variant in the segmentally duplicated *GTF2I* locus and detects methylation patterns consistent with *KDM2A*-related disorder.

**Conclusions:**

Ensilication enables diagnostic-quality native long-read sequencing without cold-chain infrastructure, supporting ambient storage and transport while preserving both sequence and methylation information.

**Supplementary Information:**

The online version contains supplementary material available at 10.1186/s13059-026-04137-4.

## Background

Long-read DNA sequencing technologies have transformed our ability to detect genetic variants and epigenetic modifications simultaneously from native DNA molecules, enabling comprehensive analysis of complex genomic regions, structural variants, and regulatory states [1]. These capabilities have proven particularly valuable for diagnosing rare genetic disorders, where critical diagnostic information inaccessible to short-read methods includes structural variants, short tandem repeats, mobile element insertions, variants in segmentally duplicated loci, and disease-specific methylation signatures [2–5]. However, the full potential of native DNA sequencing requires maintaining molecular length, sequence fidelity, and base modifications, and current best practices rely on cold-chain storage to preserve these properties.

DNA degrades through multiple pathways during storage and handling. Mechanical fragmentation reduces molecular length, hydrolytic and oxidative damage introduce sequence errors, and loss of epigenetic modifications erases regulatory information. These degradation pathways have direct diagnostic consequences. Loss of molecular length prevents resolution of structural variants in segmentally duplicated genes like *SMN1* [6–8] and *PMS2* [9], where reads must span paralogs to assign variants correctly. Sequence errors generate false positive variants that complicate clinical interpretation. Loss of native methylation patterns eliminates detection of episignatures that distinguish disorders such as Kabuki syndrome [10, 11], Fragile X syndrome [12, 13], and imprinting defects [12]. Low-temperature storage mitigates all three pathways, and clinical workflows therefore depend on cold-chain infrastructure. However, while DNA in solution can remain intact at elevated temperatures for extended periods under favorable conditions, no ambient-temperature preservation method has been systematically investigated for maintaining diagnostic-quality long-read sequencing performance across molecular length, variant calling, and methylation simultaneously. This infrastructure dependence restricts advanced genomic diagnostics to well-resourced facilities with reliable power, backup systems, and specialized shipping capability, limiting access in resource-limited settings and complicating field-based research in infectious disease surveillance and biodiversity studies.

Silica-based encapsulation has been validated for DNA preservation under extreme accelerated conditions [14–16], and a recent multi-site evaluation demonstrated diagnostic concordance between ensilicated and frozen FFPE tumor DNA using deep short-read whole-genome sequencing [17]. However, short-read library preparation includes enzymatic damage repair steps that can mask chemical modifications introduced during storage. Native long-read sequencing, which omits these repair steps, provides a more stringent test of preservation fidelity because any chemical damage inflicted by the encapsulation and release chemistry would be directly visible in the sequencing reads. Whether ensilication preserves the full information content required for native long-read sequencing, including molecular length, sequence fidelity, and epigenetic modifications at the level of individual reads, has not been evaluated. Here we show that DNA encapsulated in silica matrices and stored at ambient temperature for 30 days maintains sequencing performance equivalent to − 80 °C freezing across molecular length, sequence fidelity, and genome-wide methylation patterns. Using Genome-in-a-Bottle (GIAB) trio reference samples, we demonstrate equivalent performance between ambient-preserved and frozen DNA in read length distributions, single-read error profiles, variant calling accuracy, and methylation preservation at both the consensus and individual-read level. Ambient-preserved samples exhibited resistance to degradation during repeated handling and accelerated weathering conditions. To demonstrate clinical feasibility, we performed native long-read sequencing on ambient-preserved patient samples, resolving a de novo variant in the segmentally duplicated *GTF2I* locus and detecting the *KDM2A* methylation pattern. These results establish that diagnostic-quality native long-read sequencing is achievable without cold-chain infrastructure.

## Results

### Ensilication preserves molecular length, sequence fidelity, and genome-wide methylation patterns equivalent to −80 °C freezing

We compared DNA preservation by ensilication to conventional − 80 °C storage across molecular length, sequence fidelity, and epigenetic modifications using three GIAB reference samples (HG002, HG003, HG004). Samples were stored for 30 days under each condition and processed using Oxford Nanopore’s Native Ligation Sequencing Kit V14. Electrophoretic analysis of high-molecular-weight DNA confirmed that both frozen and ensilicated samples maintained fragment size profiles comparable to the pre-storage input (Additional file 1: Figs. S1-S3). DNA recovery after de-encapsulation and frozen sample after thawing was 90–100% of input mass as measured by Qubit fluorometry. This is consistent with recovery reported in an independent multi-site evaluation of ensilication [17].

**Fig. 1:**
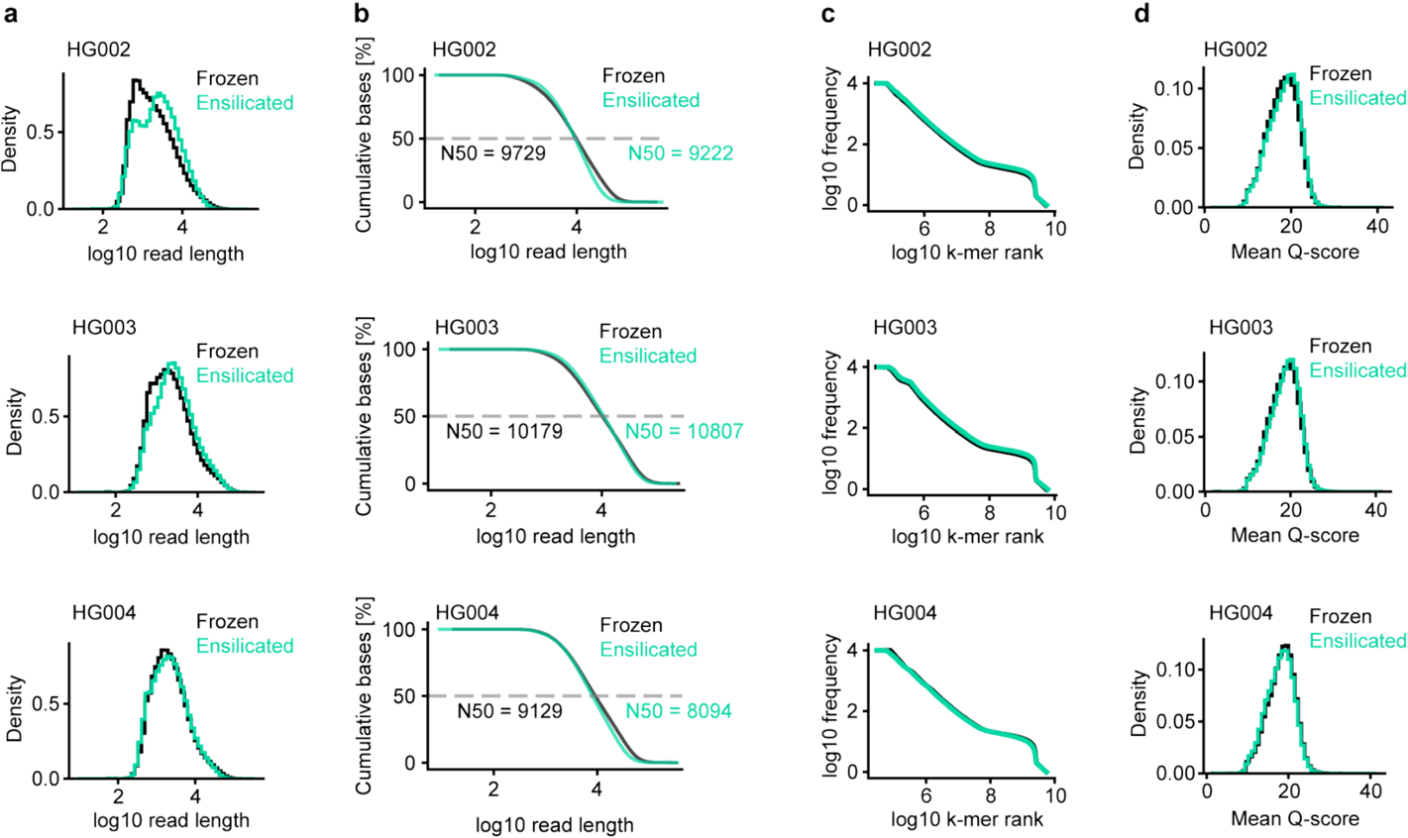
Ensilication maintains sequencing integrity comparable to conventional freezing across multiple quality metrics. **a** Read length density distributions comparing frozen (black) and ensilicated (teal) DNA preparations across three distinct GIAB reference samples: HG002, HG003, and HG004. **b** Cumulative base percentage plotted against read length, with N50 values indicated for both frozen and ensilicated conditions. **c** Log–log k-mer rank frequency distributions comparing frozen and ensilicated samples. **d** Mean Q-score density distributions demonstrating equivalent sequencing quality between frozen and ensilicated samples

Read length density distributions were closely matched between ensilicated and frozen samples across all three genomes (Fig. 1a), with N50 values ranging from 8,094 to 10,807 bp and showing no significant differences between conditions (Mann–Whitney U test, *p* = 1.0) (Fig. 1b, Table 1). Median read lengths (*p* = 0.1) and quality scores (*p* = 0.7) were also equivalent, with both methods yielding median Q-scores in the 17 to 20 range (Fig. 1d). N50 differences between conditions ranged from 507 to 1035 bp across the three samples, within the range of flowcell-to-flowcell variability typical of ONT sequencing for pre-extracted DNA samples [4]. Median read lengths were higher in ensilicated samples in all three cases (Table 1), opposite in direction to the N50 differences. This pattern is consistent with tail-driven flowcell variability rather than a systematic preservation effect on input fragment length. K-mer rank frequency analysis produced nearly identical distributions between preservation methods (Fig. 1c). At the single-read level, per-read mismatch rates (2.6–2.93%), median identity (99.00–99.08%), and the substitution spectrum were indistinguishable between conditions, with no enrichment of C > A transversions (oxidative damage) or C > T transitions (deamination) in ensilicated samples (Additional file 1: Table S1, Additional file 1: Fig. S4). Coverage uniformity was comparable, with Gini coefficients of 0.159–0.192 for frozen and 0.175–0.220 for ensilicated samples (Additional file 1: Fig. S5).

**Table 1:** Summary of sequencing metrics for frozen and ensilicated DNA samples

Genome	Storage condition	N50 [bp]	Median length [bp]	Median Q-score
HG002	Frozen	9729	1634	18.3
	Ensilicated	9222	2452	18.7
HG003	Frozen	10,807	2028	18.6
	Ensilicated	10,179	2579	18.9
HG004	Frozen	9129	2029	18.4
	Ensilicated	8094	2068	18.1

**Fig. 2:**
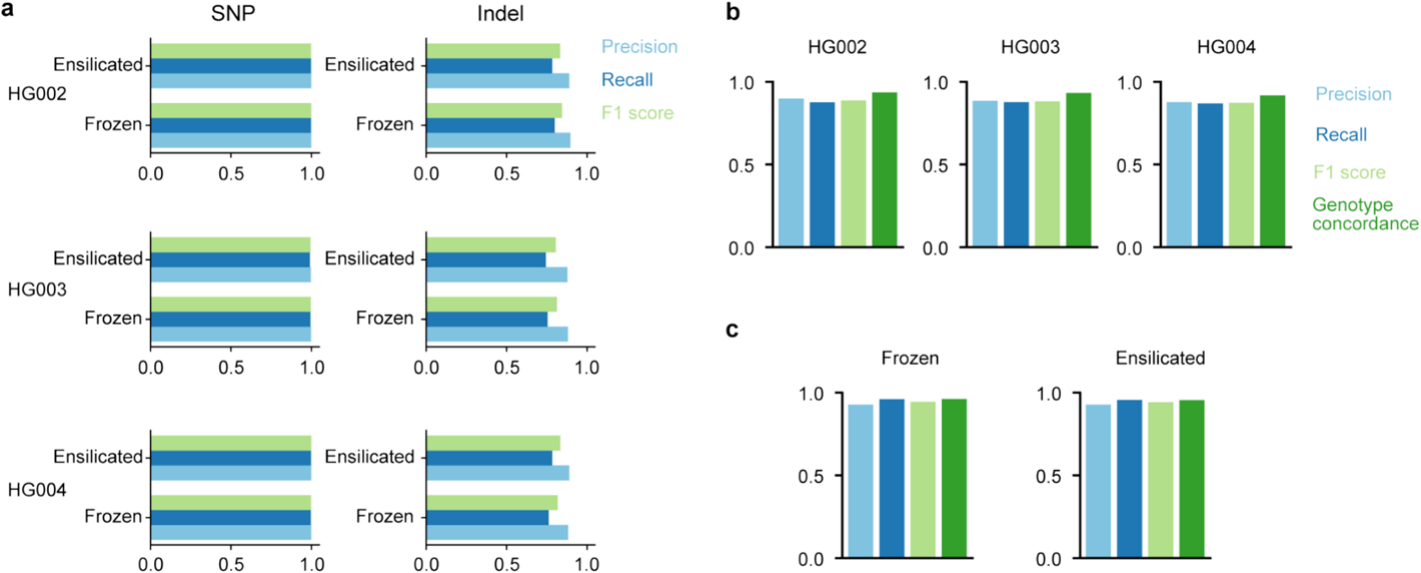
Ensilication preserves variant calling performance comparable to conventional freezing.** a** SNP (left) and indel (right) F1 scores, precision, and recall for GIAB reference samples HG002, HG003, and HG004 stored by freezing or ensilication, benchmarked against GIAB v4.2.1 small variant truth sets.** b** Structural variant concordance between ensilicated and frozen samples for all three genomes, with frozen samples used as the reference because GIAB SV truth sets are only available for HG002.** c** Structural variant performance of both ensilicated and frozen HG002 samples benchmarked independently against the GIAB Tier 1 SV truth set

Variant calling against GIAB v4.2.1 benchmarks [18] showed high concordance for both SNP and small indel detection. SNP F1 scores exceeded 0.99 across all three samples for both storage methods, and indel F1 scores were comparable between conditions (Fig. 2a). Structural variant analysis using Truvari (v4.1.0) against the GIAB Tier 1 SV truth set [19] showed precision, recall, F1 scores, and genotype concordance all exceeding 0.9 for both conditions (Fig. 2b, c). To test whether variant calling accuracy was robust to mixing preservation conditions within a family, we computed Mendelian violation rates across all eight combinations of frozen and ensilicated DNA in the HG002/HG003/HG004 trio. Violation rates ranged from 0.166% to 0.180% with no systematic difference between preservation conditions (Additional file 1: Fig. S6). The indel-SNP performance gap is characteristic of the sequencing platform rather than preservation method.

**Fig. 3:**
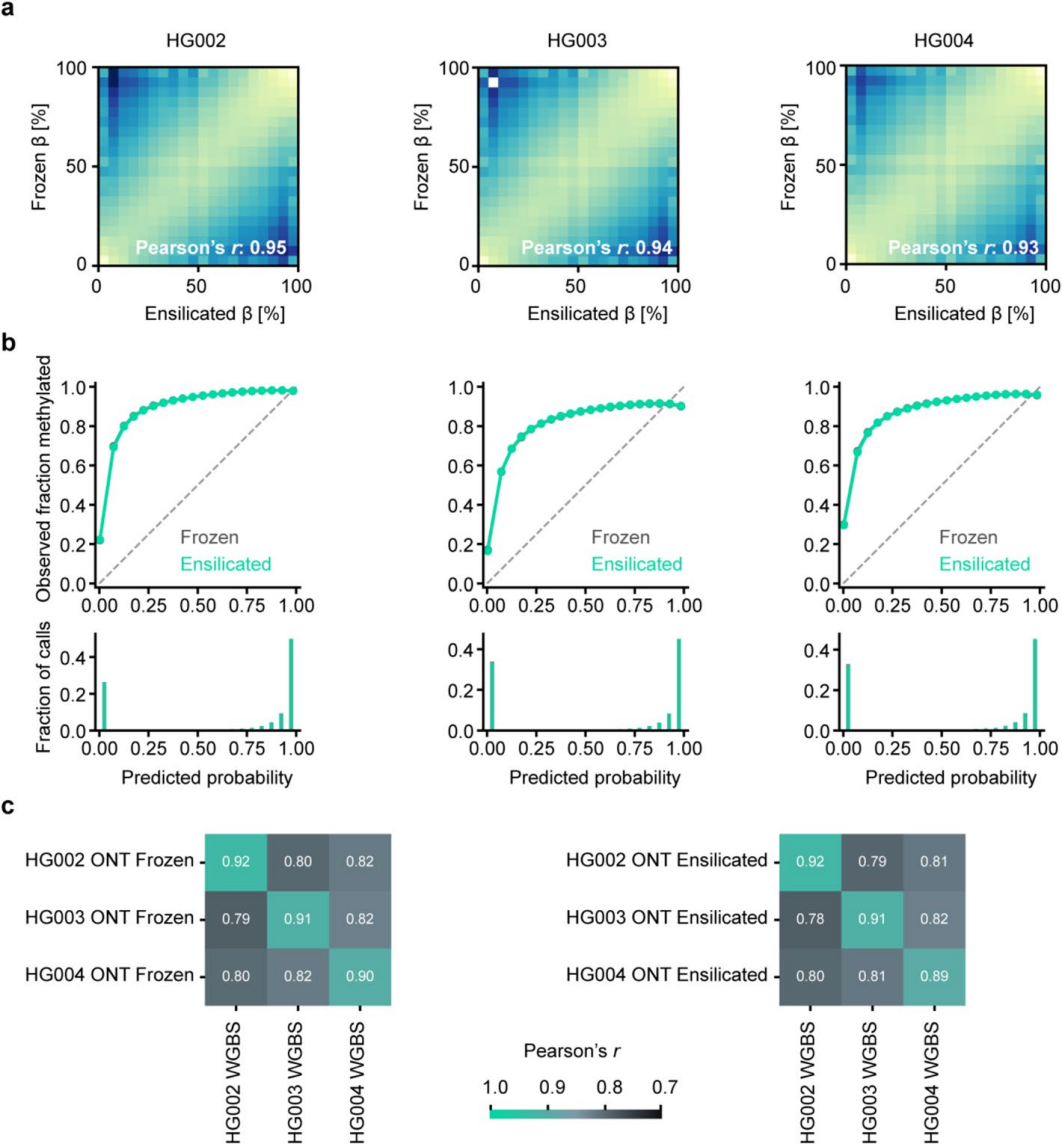
Ensilication preserves CpG methylation patterns comparable to conventional freezing.** a** Density scatter plots comparing per-CpG methylation fractions (beta values) between frozen and ensilicated samples for GIAB reference samples HG002, HG003, and HG004, with Pearson correlation coefficients indicated.** b** Methylation calibration diagrams evaluating per-read 5mC probability scores for frozen and ensilicated samples against EMSeq whole-genome bisulfite sequencing ground truth. Top panels show observed methylation fraction versus predicted probability for each sample, with the dashed diagonal representing perfect calibration. Frozen and ensilicated calibration curves are indistinguishable across the full probability range. The systematic deviation above the diagonal reflects known overestimation of methylation probability by the basecaller, independent of preservation method. Bottom panels show the distribution of predicted probabilities, confirming identical model confidence profiles between conditions.** c** Heatmaps showing Pearson correlation between ONT methylation profiles for frozen and ensilicated samples and EMSeq WGBS data from the EpiQC study [20]

Preservation of both genetic and epigenetic information is essential for comprehensive genomic analysis. Comparison of per-CpG methylation fractions (beta values) between storage conditions showed strong agreement, with Pearson correlation coefficients of 0.93 to 0.95 across all three samples (Fig. 3a). To evaluate methylation preservation at the level of individual sequencing reads rather than consensus estimates, we performed single-read ROC analysis using established EMSeq whole-genome bisulfite sequencing profiles from the EpiQC study [20] as ground truth. CpG sites were classified as methylated (EMSeq beta > = 0.80) or unmethylated (EMSeq beta < = 0.20), and individual ONT read-level 5mC calls were scored against these labels. Single-read AUC values were 0.86–0.93 across the three samples (Fig. 3b). Per-read methylation accuracy, sensitivity, and specificity were equivalent between conditions, differing by less than 0.4% across all samples and all metrics (Additional file 1: Table S2). Cross-platform correlation between ONT and EMSeq methylation profiles was also equivalent for frozen and ensilicated conditions (Pearson r > = 0.89, Fig. 3c), comparable to previously reported ONT-EMSeq concordance [20]. To assess whether the small fraction of discordant CpG sites between frozen and ensilicated conditions (6.4–10.6% of sites with |delta beta|> 0.20) reflected preservation-induced changes, we stratified discordant sites by genomic context and sequencing depth. Discordant sites were enriched in low-coverage, open-sea regions and depleted in CpG islands (Additional file 1: Tables S3-S4). A binomial sampling null model explained 85% of observed discordance, with a uniform 15% excess across all three samples consistent with technical overdispersion in ONT methylation calling rather than a preservation effect (Additional file 1: Fig. S7). At matched coverage levels, frozen and ensilicated samples showed equivalent discordance against the EMSeq reference, confirming that ensilication does not introduce measurable methylation bias beyond the inherent variability of the sequencing platform (Additional file 1: Table S5).

### Ensilication provides robust protection for ultra-long DNA under routine handling and accelerated weathering

**Fig. 4:**
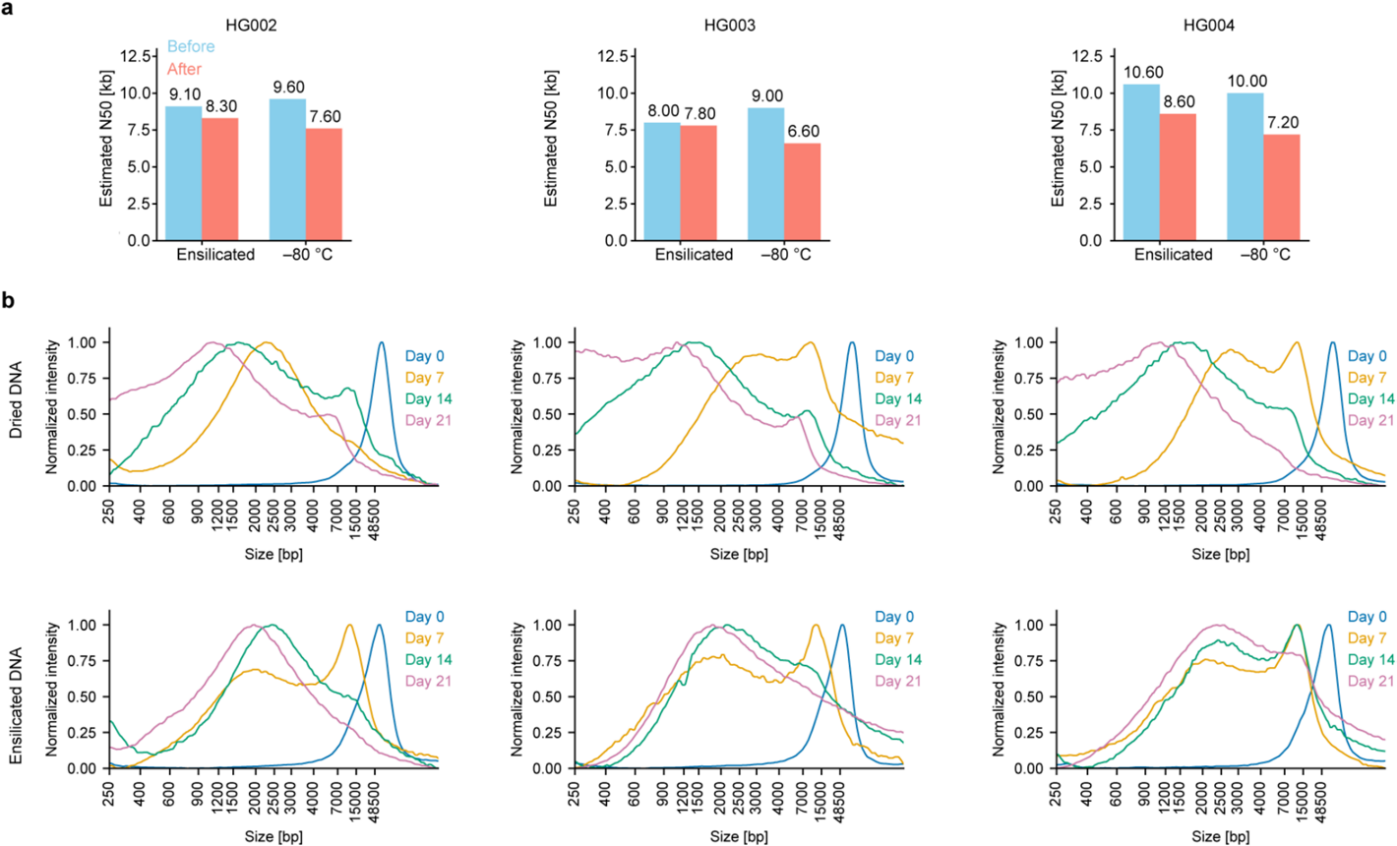
Stability of ensilicated DNA under repeated handling and accelerated weathering.** a** Estimated N50 values before and after 19 cycles of hydration-dehydration with vortexing (ensilicated) or 19 freeze–thaw cycles (− 80 °C) across GIAB reference samples HG002, HG003, and HG004.** b** Fragment size distributions of dried desalted DNA (top) and ensilicated DNA (bottom) at days 0, 7, 14, and 21 of accelerated weathering at 55 °C and 70% relative humidity

Having established that ensilication maintains DNA quality equivalent to freezing after 30 days of static storage, we next investigated whether this protection extends to conditions that more closely reflect real-world laboratory use. For frozen samples, repeated freeze–thaw cycles during routine access are unavoidable and can progressively fragment DNA. We compared the effects of 19 freeze–thaw cycles on frozen samples against 19 hydration-dehydration cycles with vortexing on ensilicated samples across all three GIAB reference genomes. N50 analysis revealed that freeze–thaw cycling reduced read lengths by 2.0–2.8 kb across the three frozen samples, while ensilicated samples subjected to hydration-dehydration stress showed smaller reductions of 0.2–2.0 kb (Fig. 4a). This difference was consistent across all three genomes, suggesting that the silica matrix provides mechanical protection during repeated sample access that is not available to DNA in solution.

To evaluate stability over longer time scales at ambient temperature, we employed an accelerated weathering protocol in which samples were incubated at 55 °C and 70% relative humidity for 21 days. Based on a previously established temperature coefficient (Q_10_ = 6.9) for DNA degradation in a similar encapsulation material [21], this exposure approximates 10,000 days of storage at 23 °C, though this extrapolation assumes consistent degradation mechanisms across temperatures. We compared ensilicated DNA against dried desalted DNA, an alternative room-temperature preservation strategy that removes water to limit hydrolytic damage. Both sample types initially displayed characteristic fragment size peaks around 48,500 bp. After 21 days of accelerated weathering, ensilicated samples maintained fragment size distributions in the 1,000–48,500 bp range, while dried DNA showed progressive fragmentation with a pronounced shift toward smaller fragments below 1000 bp (Fig. 4b). These preliminary stability data suggest that ensilication may offer advantages for long-term ambient storage, though systematic validation under diverse real-world environmental conditions is needed before definitive conclusions can be drawn.

### Ensilicated DNA supports variant resolution in structurally complex loci and episignature detection

Reference samples provide gold-standard benchmarking but represent idealized genomic contexts. Clinical diagnostics frequently require resolving variants in structurally complex regions where paralogs complicate read assignment, or interpreting variants of uncertain significance where epigenetic functional data provides critical evidence. To test whether ensilicated DNA preserves the information content necessary for such diagnostic scenarios, we performed native long-read sequencing on ambient-preserved samples from patients with rare genetic disorders.

### Validation in a segmentally duplicated locus: de novo *GTF2I* variant in a participant with autism spectrum disorder

**Fig. 5:**
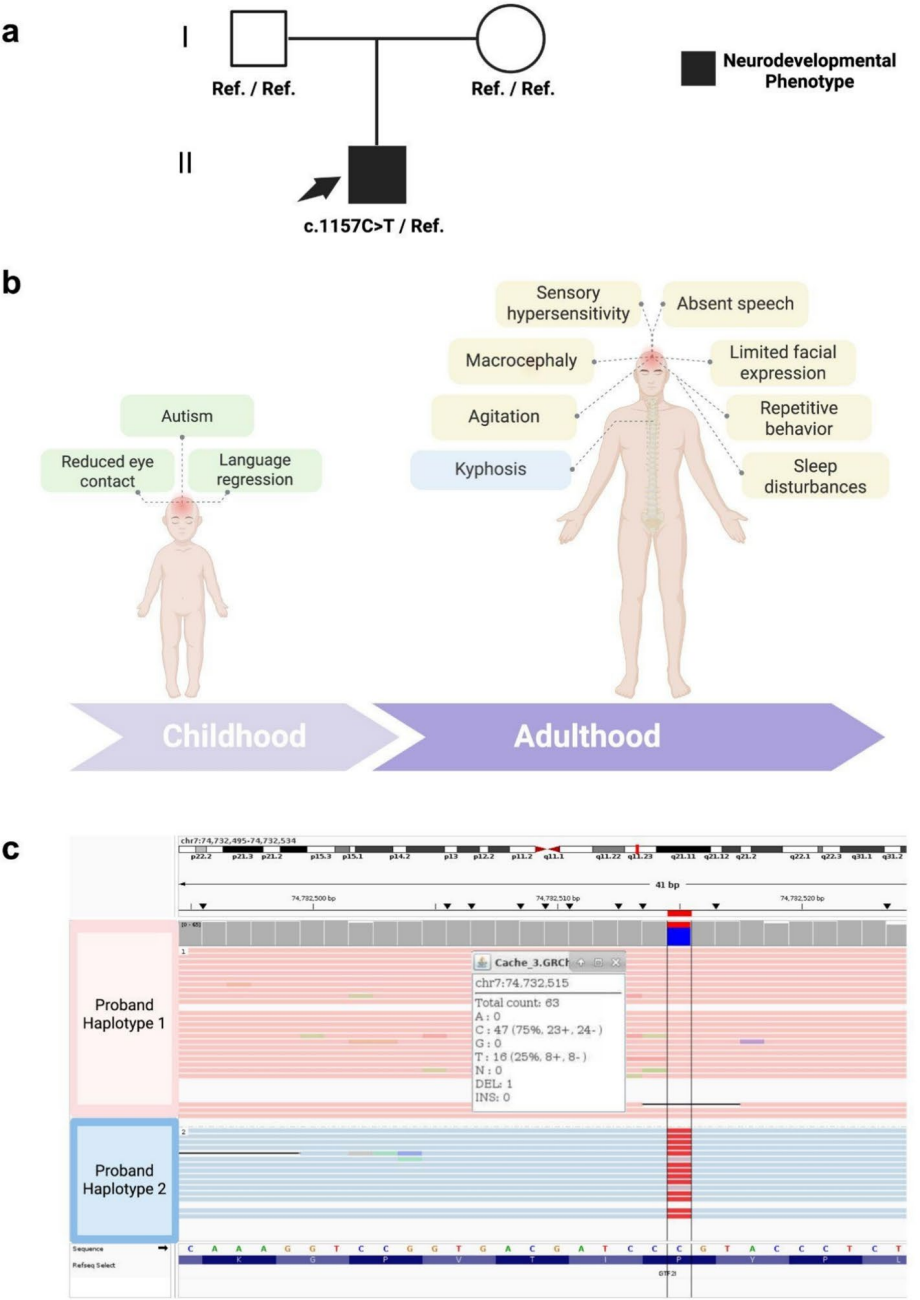
Clinical synopsis and phased long-read evidence for a heterozygous GTF2I variant. **a** Pedigree with the participant indicated by the arrow. **b** Lifespan phenotype summary. **c** IGV view of ONT native-DNA reads aligned to GRCh38 at *GTF2I* (7q11.23; vertical line at chr7:74,732,515). Reads are partitioned by phase, with Proband Haplotype 1 (top) carrying the reference base and Proband Haplotype 2 (bottom) carrying the alternate base. Red mismatches mark the alternate allele, and the inset shows base counts. The phasing confirms a single-haplotype heterozygous SNV in *GTF2I* at a segmentally duplicated locus. Created with BioRender.com

The participant is an adult male with autism spectrum disorder and intellectual disability. Language regression was noted in early childhood. Current findings include absent speech, repetitive behaviors, sensory hypersensitivity, macrocephaly, and sleep disturbances. Karyotype, Fragile X testing, and chromosomal microarray were unremarkable (Fig. 5a and b).

Clinical trio genome sequencing identified a de novo heterozygous missense variant in *GTF2I* (general transcription factor IIi, MIM [22] 601,679) (NM_032999.4:c.1157C > T; p.Pro386Leu; GRCh38 chr7:74,732,515 C > T), absent from gnomAD v4.1.0 [23, 24] and predicted to be deleterious (CADD [25] 28). This variant is listed in ClinVar [26] as of uncertain significance (VCV003359229.2). *GTF2I* is located within the Williams-Beuren syndrome critical region (WBS, MIM [22] 194,050). Because *GTF2I* resides within the 7q11.23 segmental duplication block and has a closely related paralog/pseudogene (*GTF2IP1*), accurate read assignment is a known challenge [27]. To verify locus specificity, we used ONT long-read sequencing. ONT reads covering unique paralog-distinguishing motifs confirmed the variant’s position (read depth of 63 ×, 16 alternate reads (VAF 0.25)) in *GTF2I* (not *GTF2IP1*) (Fig. 5c), with phasing consistent with heterozygosity and trio short-read genome data supporting a de novo origin. EpiSign [28] methylation profiling revealed a mildly abnormal pattern that partially overlapped with the Williams–Beuren syndrome epigenome signature; however, the overall results were inconclusive and negative for other conditions tested. In future analyses of this case, we plan to evaluate ONT-derived methylation signals using newer long-read classifiers to enable quantitative episignature interpretation.

Recent evidence supports a monogenic *GTF2I*-related neurodevelopmental disorder [27]. A multicenter case series identified eight unrelated individuals with heterozygous *GTF2I* alterations, including de novo nonsense, splice-site, missense, in-frame deletion, and intragenic deletion variants, as well as one case with reduced *GTF2I* expression. Affected individuals shared global developmental delay or intellectual disability with frequent autistic features and facial dysmorphism partly reminiscent of Williams–Beuren syndrome. RNA sequencing confirmed aberrant splicing or decreased expression for several variants, consistent with a haploinsufficiency mechanism. The study also highlighted technical challenges due to homology with *GTF2IP1* and demonstrated that long-read sequencing can aid in variant confirmation. These findings provide human genetic validation that deleterious *GTF2I* variation can underlie isolated neurodevelopmental disease and align with prior functional models of *GTF2I* dosage sensitivity [29].

### Genetic variant confirmation and methylation changes detection in a *KDM2A* case

The participant is a pre-adolescent female evaluated for dysmorphic features, generalized hypotonia, global development delay, feeding difficulties with early failure to thrive, autism spectrum disorder, and attention-deficit/hyperactivity disorder with behavioral dysregulation. Prior chromosomal microarray, exome sequencing, and genome sequencing were nondiagnostic.

**Fig. 6:**
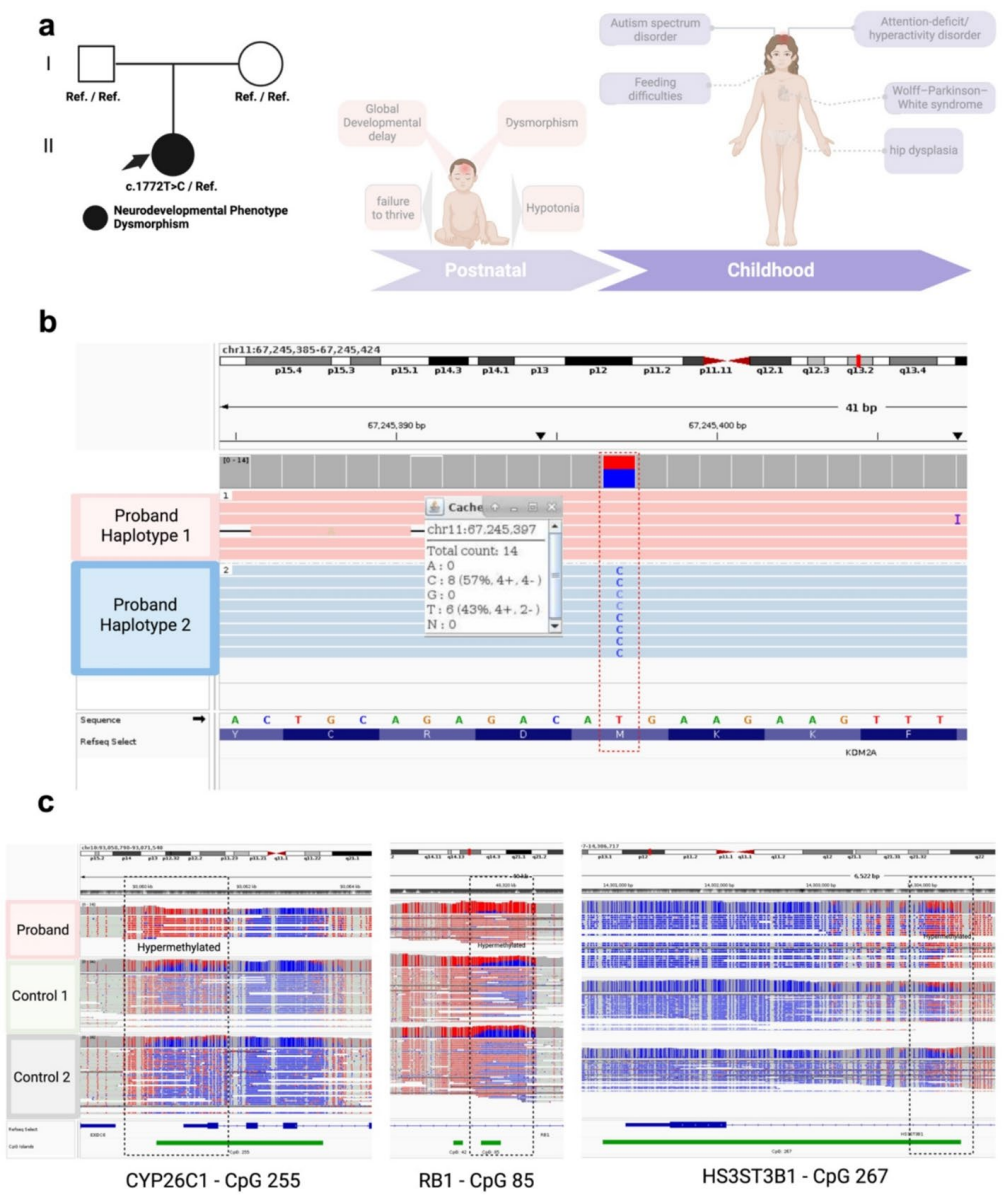
Clinical course, haplotype-resolved confirmation, and representative DNA-methylation CpG islands for a *KDM2A* variant.** a** Pedigree and phenotype timeline. The participant (arrow, filled symbol) carries a de novo *KDM2A* c.1772 T > C variant (parents Ref./Ref.) and presents with neurodevelopmental features consistent with *KDM2A*-related disorder. **b** IGV view of Oxford Nanopore native-DNA reads aligned to GRCh38 at *KDM2A* (chr11:67,245,397). Reads are partitioned by phase, with Proband Haplotype 1 carrying the reference allele and Haplotype 2 carrying the alternate allele. Base counts support a heterozygous single-nucleotide variant present on a single haplotype. **c,** Representative native-DNA methylation tracks at three CpG sites showing methylation changes suggestive of *KDM2A*-related disorder (CYP26C1 CpG255, RB1 CpG85, HS3ST3B1 CpG267). The participant exhibits focal hypermethylation relative to two controls (IGV convention: red, methylated; blue, unmethylated; dashed boxes indicate comparison windows), providing orthogonal functional support for the *KDM2A* variant. Created with BioRender.com

Genetic re-analysis identified a de novo heterozygous *KDM2A* (lysine demethylase 2 A, MIM [22] 605,657) variant, NM_012308.3:c.1772 T > C (p.Met591Thr), confirmed by Sanger sequencing. This participant was included in a *KDM2A* cohort study [30], establishing its clinical relevance within a syndromic neurodevelopmental disorder. Using ONT long-read sequencing, we confirmed the variant’s position (read depth of 14 ×, 8 alternate reads (VAF 0.57)). We then used native DNA cytosine methylation calls from the same ONT dataset to visualize representative loci implicated in the *KDM2A* methylation signature and compared the proband qualitatively with ambient-preserved control specimens without *KDM2A* variants (Fig. 6). The proband exhibited hypermethylated CpG islands, including CYP26C1 CpG255, RB1 CpG85, and HS3ST3B1 CpG267. The qualitative pattern aligned with the *KDM2A*-positive cluster described in the cohort analysis [30]. Due to the limited scope of our ONT subset, these methylation findings are presented descriptively to demonstrate feasibility rather than to provide classifier-grade output. Future work could leverage emerging ONT-based methylation classifiers to enable quantitative classification [31, 32].

## Discussion

Our results demonstrate that ensilication preserves DNA at ambient temperature for 30 days with sequencing performance equivalent to conventional − 80 °C freezing across molecular length, variant calling, and genome-wide methylation. This equivalence holds at both the consensus level and at the level of individual sequencing reads. Per-read mismatch rates, substitution spectra, and single-read methylation accuracy were indistinguishable between preservation conditions across all three GIAB reference genomes. Mendelian concordance analysis across all combinations of frozen and ensilicated samples in the HG002/HG003/HG004 trio confirmed that preservation method does not introduce genotyping errors detectable through inheritance-based validation.

The methylation results warrant detailed consideration because native epigenetic information is among the most sensitive indicators of sample integrity and because consensus-level comparisons can mask read-level differences. We therefore evaluated methylation preservation at the single-read level against an independent bisulfite sequencing reference rather than using frozen ONT data as ground truth. Per-read 5mC calling accuracy differed by less than 0.4% between frozen and ensilicated conditions across all three samples, and single-read ROC AUC values were indistinguishable at the precision reported. Discordant CpG sites between conditions were concentrated in low-coverage, open-sea regions and were largely explained by a binomial sampling null model, with a uniform 15% excess across all three samples attributable to technical overdispersion in ONT methylation calling rather than a preservation-specific effect. At matched coverage levels, frozen and ensilicated samples showed equivalent discordance against the EMSeq reference, confirming that any residual methylation differences reflect the inherent variability of the sequencing platform rather than preservation-induced changes.

Analysis of the substitution spectrum provided direct evidence that ensilication does not introduce chemical damage to DNA. Oxidative damage characteristically elevates C > A transversions through 8-oxoguanine formation, and cytosine deamination elevates C > T transitions. Neither signature was enriched in ensilicated samples relative to frozen controls. This finding is consistent with the proposed mechanism of silica encapsulation, in which the low-water-activity microenvironment restricts hydrolytic and oxidative access to the nucleobases.

Beyond static storage, ensilicated DNA showed greater resilience than frozen DNA to repeated handling, with smaller N50 reductions after 19 access cycles across all three genomes. This has practical implications for biobanking workflows where samples must be accessed multiple times without aliquoting. Accelerated weathering experiments further suggested long-term ambient stability, though the extrapolation from 21 days at 55 °C to years of room-temperature storage rests on assumptions about consistent degradation mechanisms across temperatures that require empirical validation through real-world longitudinal studies.

The clinical cases demonstrate that ambient-preserved DNA supports variant resolution in a segmentally duplicated locus and detection of disease-associated methylation patterns in patients with rare genetic disorders. These cases establish feasibility rather than a controlled comparison between preservation methods. A definitive assessment of clinical equivalence would require parallel sequencing of the same patient samples preserved under multiple conditions, which was beyond the scope of this study.

Several limitations should be noted. The present study evaluated ensilication using high-molecular-weight genomic DNA from lymphoblastoid cell lines and two clinical blood-derived samples. An independent study demonstrated that ensilication also preserves FFPE tumor DNA with complete diagnostic concordance across matched tumor-normal pairs, and that frozen FFPE samples accumulated more artifactual C > T mutations than ensilicated samples, likely due to continued hydrolysis by co-stored fixation byproducts [17]. Extension to additional sample types including fresh tissue, cell-free DNA, and microbial communities will be necessary to establish broader applicability. The 30-day ambient storage period, while sufficient to demonstrate equivalence to freezing, does not address the multi-year timescales relevant to biobanking. Long-term stability studies under varied temperature, humidity, and handling conditions will be needed to establish performance boundaries. We did not compare ensilication against DNA stored in solution at ambient temperature. Such a comparison is not straightforward, as aqueous storage introduces confounding variables at both extremes. At elevated temperatures used in accelerated weathering, evaporation alters solute concentrations in ways that can accelerate degradation. At cryogenic temperatures, freeze concentration at ice crystal boundaries creates localized microenvironments with elevated solute and DNA concentrations that may promote damage through mechanisms distinct from bulk solution chemistry [17]. These confounds highlight the difficulty of establishing a clean aqueous baseline and underscore the advantage of a preservation method that removes DNA from solution entirely. Real-time comparisons at room temperature over extended periods would avoid these artifacts and are an important direction for future work.

## Conclusions

Ensilication preserves DNA integrity at ambient temperature for 30 days equivalent to conventional −80 °C freezing across molecular length, sequence fidelity, and genome-wide methylation patterns, as demonstrated at both the consensus and single-read level. Ambient-preserved DNA supports diagnostic-quality variant calling in structurally complex loci and detection of disease-associated methylation patterns. Systematic validation across longer time scales, diverse environmental conditions, and additional sample types will define the boundaries of this approach and inform practical implementation. Beyond logistics, ambient DNA preservation has implications for how we approach genomic data management. Preserving the physical molecule at room temperature may be more economical than long-term digital storage of raw sequencing data, with the added advantage that preserved samples can be re-interrogated as assay platforms improve rather than committing to a single technological snapshot [33]. As sequencing platforms continue to become more portable and accessible, the geographic scope of genomic medicine need no longer be strictly defined by infrastructure limitations in sample preservation.

## Methods

### Study cohort

We evaluated two participants enrolled in the Undiagnosed Diseases Network (UDN) using Oxford Nanopore Technologies (ONT) long-read whole-genome sequencing (lrWGS) from whole-blood DNA: Case 1 with a candidate variant in *GTF2I*, and Case 2 with a research-solved candidate in *KDM2A* that we used as a positive control for our post-encapsulation workflow.

### Genomic DNA encapsulation and library preparation

Genomic DNA (3 µg) was dissolved in 1000 µL nuclease-free water (Thermo Fisher; catalog number: AM9916) in a 1.5 mL Eppendorf LoBind tube. Encapsulation particles from Cache DNA, Inc. (3 mg) were added, and the suspension was mixed by turning the tube upside down 3 times. Ensilication reagents from Cache DNA, Inc. were then added, and the mixture was shaken for 48 h at 1600 rpm using a Bioshake iQ (Bulldog Bio; catalog number: 1808–0506).

The suspension was pelleted with a microcentrifuge for 10 s and the supernatant was carefully removed. The pellet was washed with 0.1% Tween-20 and stored for 30 days at ambient temperature until further processing. For DNA retrieval, 300 µL of de-encapsulation buffer (Cache DNA, Inc.) was added, and the mixture was incubated at ambient temperature for 5 min. De-encapsulated DNA was purified using a Multi-screen 96-well filter plate (Millipore Sigma; catalog number: MSNU03010). Briefly, 300 µL of the sample was added to a well and filtered to dryness. A volume of 150 µL of nuclease-free water was then added and also filtered to dryness. Finally, a volume of 55 µL of resuspension buffer (20 mM Tris + 0.01% Tween-20) was added, and the sample was mixed for 5 min on a Bioshake iQ shaker. The purified DNA was used directly for library preparation with the Ligation Sequencing Kit V14 (Oxford Nanopore Technologies; catalog number: SQK-LSK114) following the manufacturer's protocol. Access to ensilication and de-encapsulation reagents can be addressed to Cache DNA, Inc.

### Nanopore sequencing

Genomic DNA libraries were prepared using the Oxford Nanopore Ligation Sequencing Kit V14 (SQK‑LSK114) on PromethION with R10.4.1 flow cells (FLO‑PRO114M). Three µg of high‑molecular‑weight DNA were transferred to a 1.5 mL DNA LoBind tube and brought to 47 µl with nuclease‑free water (Thermo Fisher Scientific, Cat. 10,977,015). DNA repair and end‑prep were performed in a 0.2 mL PCR tube with the NEBNext Companion Module for Oxford Nanopore Ligation Sequencing (NEB, Cat. E7180L) following the manufacturer’s instructions, incubating at 20 °C for 5 min and 65 °C for 5 min, then cooling to 4 °C on a thermocycler. The reaction was purified by SPRI using AMPure XP on a magnetic rack with two 80% ethanol washes and eluted in 61 µl nuclease‑free water (Beckman Coulter, Cat. A63881). Sequencing adapters (LA) were ligated in ONT Ligation Buffer (LNB) with NEB Quick T4 DNA Ligase from the module, followed by clean‑up using ONT Long Fragment Buffer (LFB) to enrich fragments ≥ 3 kb and elution in 25 µl Elution Buffer (EB). Library yield was quantified fluorometrically (Qubit Fluorometer with dsDNA kit, Thermo Fisher Scientific, Cat. Q32853) and supported multiple reloads per flow cell. For loading, 32 µl of library were combined with Sequencing Buffer (SB) and Library Solution (LIS) to a final 200 µl mix. Flow cells were primed with Flow Cell Flush (FCF) and Flow Cell Tether (FCT), and 200 µl of the library were loaded per the manufacturer’s recommendations. Sequencing was run on a PromethION 48 under MinKNOW control for 72 h; the run was paused at 24 h, the flow cell washed (Flow Cell Wash Kit, EXP‑WSH004) and reloaded, then resumed.

### Genome-in-a-Bottle small variant benchmarking

Small variants (SNVs and indels) were called from each aligned BAM using DeepVariant v1.6.0 (docker image google/deepvariant:1.6.0) with the ONT_R104 model and GPU acceleration, parallelized across 32 shards. Variant calls were benchmarked against GIAB v4.2.1 truth sets [18] for each sample (HG002, HG003, HG004) using hap.py v0.3.12 (docker image jmcdani20/hap.py:v0.3.12) with the vcfeval engine (RTG Tools). Evaluation was restricted to GIAB high-confidence regions (HG00{2,3,4}_GRCh38_1_22_v4.2.1_benchmark_noinconsistent.bed) with –pass-only, counting only PASS-filtered variant calls. Hap.py decomposes complex variants and performs haplotype-aware comparison, classifying each call as true positive, false positive, or false negative, from which precision, recall, and F1 score were computed separately for SNVs and indels. Additional benchmarking was performed for HG002 against the CMRG small variant benchmark v1.00 (HG002_GRCh38_CMRG_smallvar_v1.00) and the CMRG tandem repeat benchmark v1.0 (HG002_GRCh38_TandemRepeats_v1.0).

### Structural variant benchmarking

Structural variants were called from each aligned BAM using Sniffles2 v2.2 with default parameters. SV calls were benchmarked for HG002 against the GIAB Tier 1 SV truth set v0.6 (HG002_SVs_Tier1_v0.6.vcf.gz) [19] and corresponding high-confidence regions (HG002_SVs_Tier1_v0.6.bed) using Truvari v4.1.0. Truvari matching parameters were: maximum reference distance 500 bp (–refdist 500), minimum size 50 bp (–sizemin 50), size filter 30 bp (–sizefilt 30), maximum size 50,000 bp (–sizemax 50,000), minimum size similarity 70% (–pctsize 0.7), sequence similarity disabled (–pctseq 0.0), reciprocal overlap disabled (–pctovl 0.0), PASS-only calls (–passonly). Additional SV benchmarking was performed against the CMRG SV benchmark v1.00 (HG002_GRCh38_CMRG_SV_v1.00).

### Single-read methylation calibration

Per-read 5mC probabilities were evaluated against an EMSeq consensus truth set constructed for each GIAB sample. EMSeq bedGraph files were filtered to autosomal and chrX CpG sites with total coverage > = 10 reads. Sites with EMSeq beta > = 0.80 were labeled methylated (truth = 1), sites with beta < = 0.20 were labeled unmethylated (truth = 0), and intermediate sites were excluded. For each ONT BAM (Dorado v1.4.0, aligned with minimap2 v2.28, -ax map-ont -y -K5g), per-read 5mC probabilities were extracted from the MM and ML auxiliary tags using pysam (v0.21.0) across all primary, non-supplementary, mapped alignments on autosomes and chrX. For each modified CpG position within a read, the ML tag probability (unsigned 8-bit integer, 0–255) was mapped to a reference coordinate via get_reference_positions(full_length = True) and matched to the EMSeq truth set. ML probabilities were normalized to [0, 1] by dividing by 255 and binned into 20 equal-width intervals. Within each bin, the mean predicted probability and observed fraction methylated (fraction of observations with truth label = 1) were computed. Bins with fewer than 100 observations were excluded. Per-read accuracy, sensitivity, and specificity were computed by binarizing ML probabilities at a threshold of 128 (*p* > 0.50 = methylated).

### Pairwise Pearson correlation matrix of CpG methylation

Per-CpG methylation values were obtained for nine datasets: GIAB trio samples (HG002, HG003, HG004) each assayed by three methods (ONT frozen, ONT ensilicated, and EMseq WGBS). ONT methylation was called by modkit v0.3 pileup with –combine-strands –cpg –bedgraph, which reports the fraction of reads carrying the 5mC modification at each CpG dinucleotide (beta, range 0–1) and the total read depth. EMseq methylation was obtained from ENCODE/GIAB bedGraph files reporting methylation percentage (0–100), methylated read count, and unmethylated read count per CpG site; the percentage was divided by 100 to convert to the 0–1 beta scale. For each of the 36 unique pairwise combinations of the nine datasets, the two bedGraph files were inner-joined on genomic position (chromosome, start, end) using pandas v2.2.2, retaining only CpG sites present in both files with no minimum coverage filter at this stage. The Pearson product-moment correlation coefficient was then computed between the two beta-value vectors at the overlapping sites using scipy v1.13.1 pearsonr, which returns both the correlation coefficient r and the two-sided *p*-value testing the null hypothesis that the two vectors are uncorrelated. Specifically, for paired beta values (x_i_, y_i_) across n overlapping sites, r = sum[(x_i_—x_mean_)(y_i_—y_mean_)]/sqrt[sum(x_i_—x_mean_)^2^ × sum(y_i_—y_mean_)^2^]. The resulting r values were assembled into a symmetric 9 × 9 matrix with 1.0 along the diagonal.

### Variant and methylation review

Loci of interest (*GTF2I*, *KDM2A*) were inspected in IGV [34] (version 2.19.2). CpG 5mC tracks were generated from native signals using the study pipeline and viewed alongside CpG-island annotations. For each locus, we report local coverage, variant-supporting read counts, approximate variant allele fraction, and the presence or absence of nearby structural variants within the inspected window.

### Exploratory methylation analysis

To illustrate the *KDM2A* episignature in native nanopore data, we visualized representative sites previously implicated in the signature (for example, CYP26C1 CpG255, RB1 CpG85, and HS3ST3B1 CpG267) and compared the proband qualitatively against ensilicated controls without *KDM2A* variants. Given the small sample size, these analyses are descriptive.

## Supplementary Information

Additional file 1: Supplementary figures and tables. Contains the following items. Fig. S1. TapeStation traces of genomic samples as-received. Fig. S2. TapeStation traces of frozen genomic samples after 30 days of storage prior to library preparation. Fig. S3. TapeStation traces of ensilicated genomic samples after 30 days of storage prior to library preparation. Fig. S4. Substitution spectra for frozen and ensilicated samples across the GIAB trio, showing the relative frequency of the six canonical single-nucleotide substitution types. Fig. S5. Lorenz curves of per-position read depth uniformity for frozen and ensilicated samples across HG002, HG003, and HG004, with Gini coefficients indicated. Fig. S6. Heatmaps of Mendelian violation rates across all eight combinations of frozen and ensilicated DNA in the GIAB trio. Fig. S7. Methylation discordance analysis. Genome-wide scatterplots of per-CpG methylation fractions between frozen and ensilicated samples (left), observed versus expected discordance as a function of coverage under a binomial sampling null model (middle), and distributions of absolute methylation differences with the binomial null expectation overlaid (right). Table S1. Genome-wide per-read sequencing error profiles for frozen and ensilicated samples across HG002, HG003, and HG004, including mismatch rate, median identity, and supplementary alignment rate. Table S2. Single-read methylation concordance against EMSeq ground truth for frozen and ensilicated samples across HG002, HG003, and HG004, including accuracy, sensitivity, and specificity. Table S3. CpG context distribution and discordance rates by genomic context (island, shore, shelf, open sea) for frozen versus ensilicated methylation comparisons. Table S4. Local GC content and coverage of concordant versus discordant CpG sites for frozen versus ensilicated methylation comparisons. Table S5. Coverage-stratified methylation discordance rates against EMSeq for frozen and ensilicated samples across HG002, HG003, and HG004.

## Data Availability

Raw nanopore sequencing data for GIAB reference samples (HG002, HG003, HG004) under frozen and ensilicated conditions have been deposited in the NCBI Sequence Read Archive under BioProject accession PRJNA1445941 [35] Sequencing data from patients enrolled in the Undiagnosed Diseases Network (UDN) are made available through dbGaP (phs001232.v7.p3) [36] to qualified investigators. Analysis scripts for variant calling benchmarking, single-read methylation calibration, substitution spectrum analysis, Mendelian violation analysis, binomial null modeling of methylation discordance, and coverage uniformity assessment are available at github.com/jearlbcache/fossilized-ark [37] and Zenodo [38]. Source code is released under the MIT license. GIAB truth sets (v4.2.1 [39] small variant, Tier 1 SV v0.6 [40]) are available from https://ftp-trace.ncbi.nlm.nih.gov/ReferenceSamples/giab/. EMSeq whole-genome bisulfite sequencing data from the EpiQC study are available under the accessions described in the original publication [41]. The ensilication and de-encapsulation reagents are commercially available from Cache DNA, Inc. The patents covering the technology do not restrict academic research use of the reagents, independent reproduction of the analyses described in this study, or evaluation of ensilicated samples using methods other than those proprietary to Cache DNA, Inc.
