# Supplementary material for: Ensilication preserves high-molecular weight native DNA for clinical long-read sequencing
Source: Genome Biol. 2026 Jun 15;27:194. doi: 10.1186/s13059-026-04137-4 (PMC13267750; doi:10.1186/s13059-026-04137-4)
Supplement: Supplementary file 1 — Additional file 1: Supplementary figures and tables. Contains the following items. Fig. S1. TapeStation traces of genomic samples as-received. Fig. S2. TapeStation traces of frozen genomic samples after 30 days of storage prior to library preparation. Fig. S3. TapeStation traces of ensilicated genomic samples after 30 days of storage prior to library preparation. Fig. S4. Substitution spectra for frozen and ensilicated samples across the GIAB trio, showing the relative frequency of the six canonical single-nucleotide substitution types. Fig. S5. Lorenz curves of per-position read depth uniformity for frozen and ensilicated samples across HG002, HG003, and HG004, with Gini coefficients indicated. Fig. S6. Heatmaps of Mendelian violation rates across all eight combinations of frozen and ensilicated DNA in the GIAB trio. Fig. S7. Methylation discordance analysis. Genome-wide scatterplots of per-CpG methylation fractions between frozen and ensilicated samples (left), observed versus expected discordance as a function of coverage under a binomial sampling null model (middle), and distributions of absolute methylation differences with the binomial null expectation overlaid (right). Table S1. Genome-wide per-read sequencing error profiles for frozen and ensilicated samples across HG002, HG003, and HG004, including mismatch rate, median identity, and supplementary alignment rate. Table S2. Single-read methylation concordance against EMSeq ground truth for frozen and ensilicated samples across HG002, HG003, and HG004, including accuracy, sensitivity, and specificity. Table S3. CpG context distribution and discordance rates by genomic context (island, shore, shelf, open sea) for frozen versus ensilicated methylation comparisons. Table S4. Local GC content and coverage of concordant versus discordant CpG sites for frozen versus ensilicated methylation comparisons. Table S5. Coverage-stratified methylation discordance rates against EMSeq for frozen and ensilicated samples acros [file 13059_2026_4137_MOESM1_ESM.pdf]

Additional File 1:

Ensilication preserves high-molecular weight native DNA for clinical long-read sequencing

Alexis Ferrasse[1,6], Rodrigo Mendez[1,2,6], John E. Gorzynski[1,2,3,6], Chloe Reuter[1,2], Jennefer N. Carter[1,2], Michael Blas[5], Undiagnosed Diseases Network, Jonathan A. Bernstein[2,4], Matthew T. Wheeler[1,2], James L. Banal[5,7], Euan A. Ashley[1,7]

[1] Stanford University, Department of Medicine, Division of Cardiovascular Medicine, Stanford, CA, USA

[2] Stanford Center for Undiagnosed Diseases, Stanford, CA, USA

[3] Stanford University School of Medicine, Department of Genetics, Stanford, CA, USA

[4] Division of Medical Genetics, Department of Pediatrics, Stanford University School of Medicine, Stanford, CA

[5] Cache DNA, Inc. San Carlos, CA, USA

[6] These authors contributed equally to this work

[7] Corresponding author: james@cache-dna.com and euan@stanford.edu

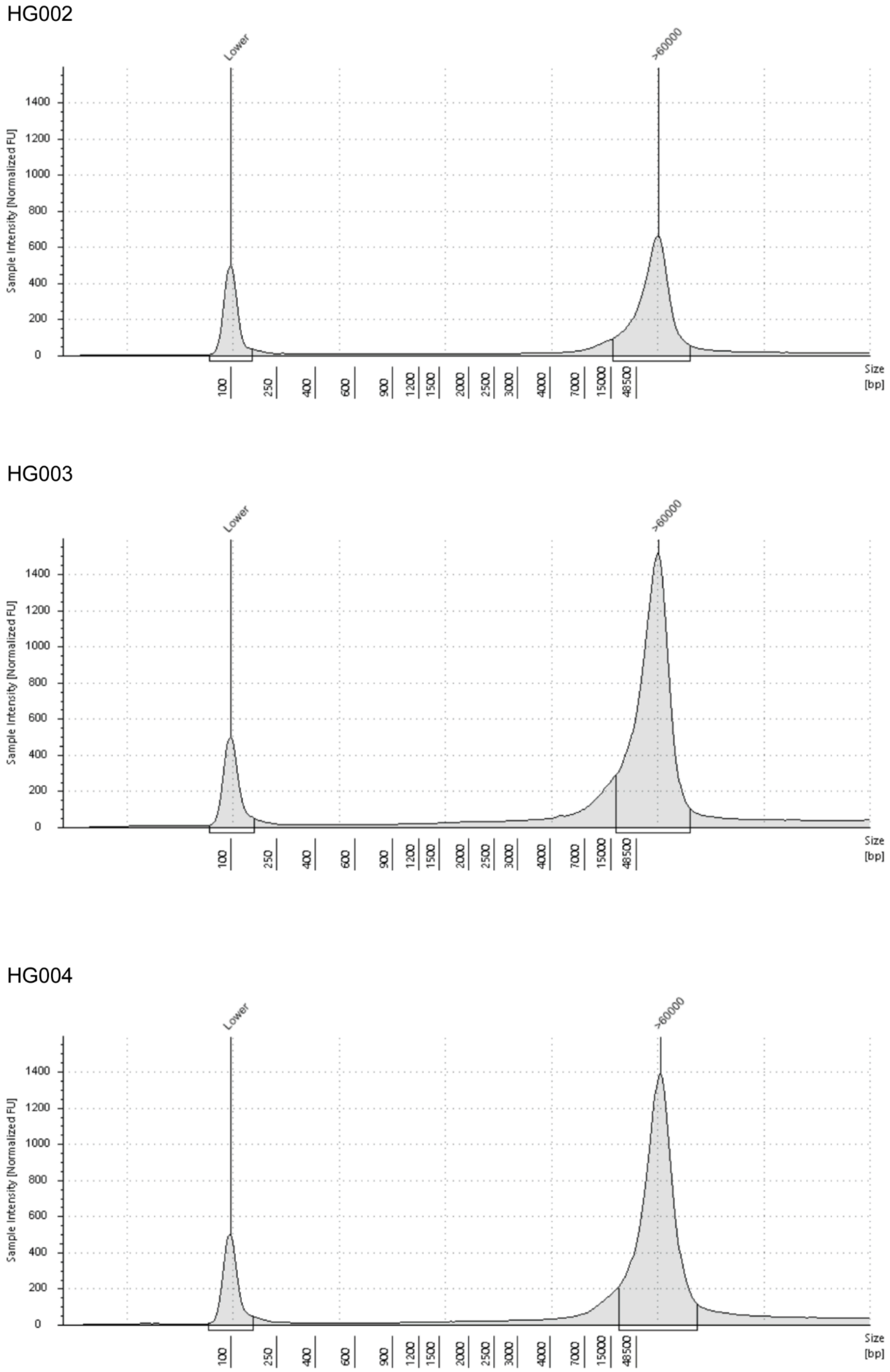

Fig. S1. Tapestation traces of genomic samples as-received.

HG002

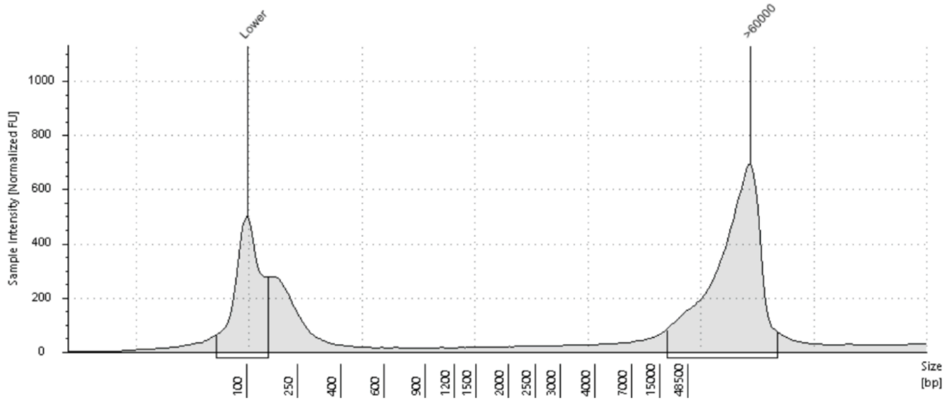

HG003

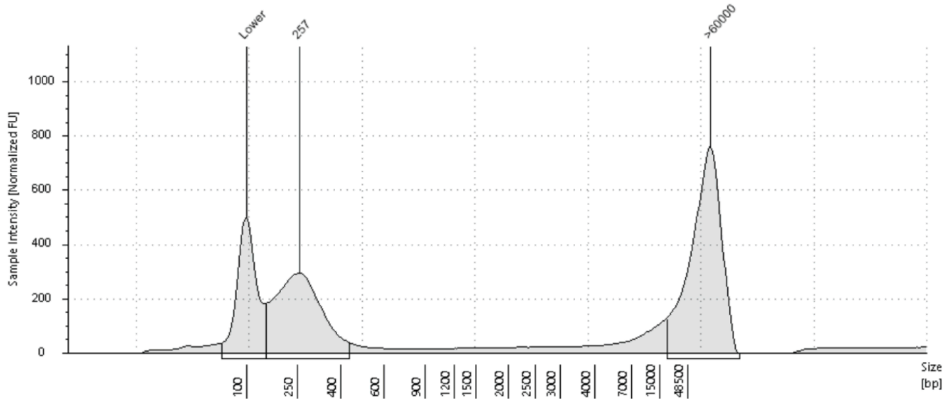

HG004

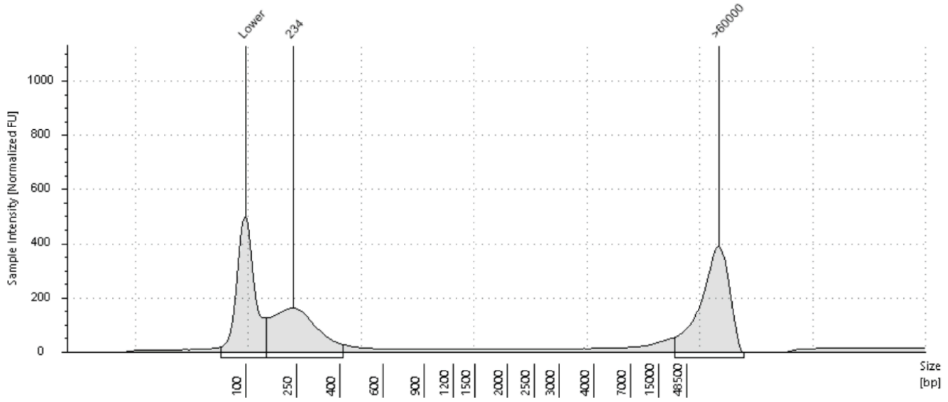

Fig. S2. Tapestation traces of frozen genomic samples after 30 days of storage prior to library preparation.

HG002

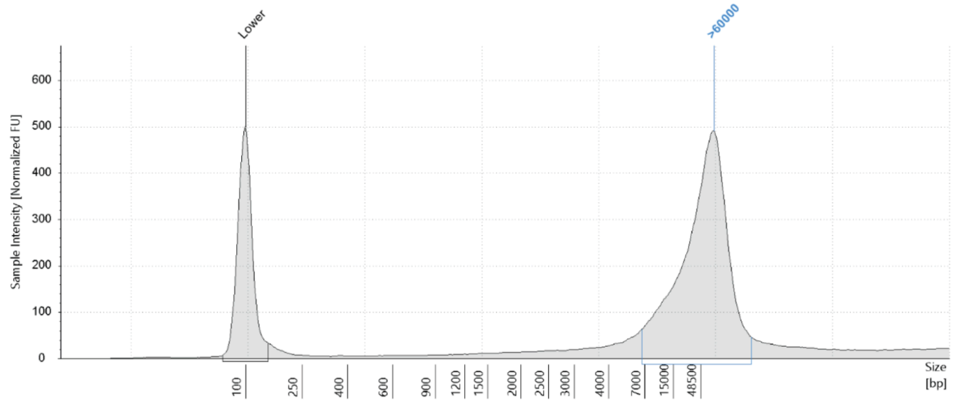

HG003

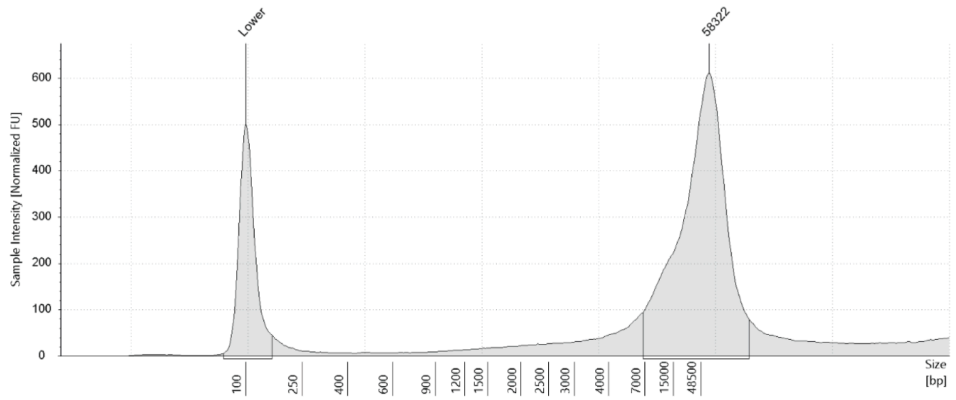

HG004

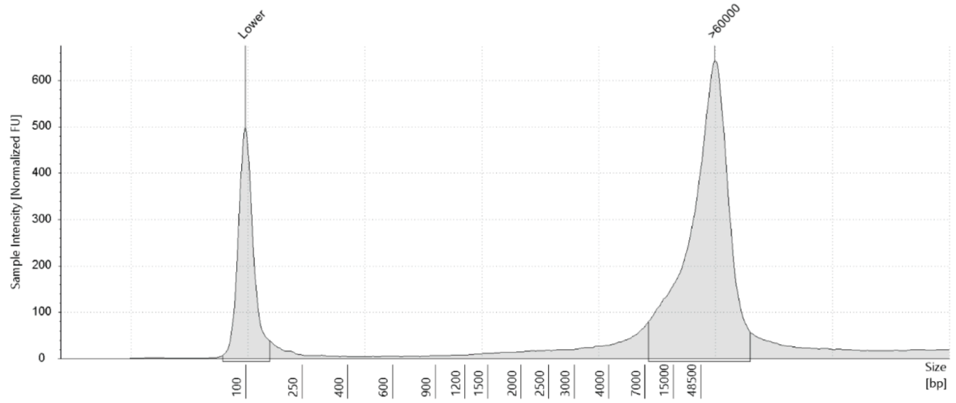

Fig. S3. Tapestation traces of ensilicated genomic samples after 30 days of storage prior to library preparation.

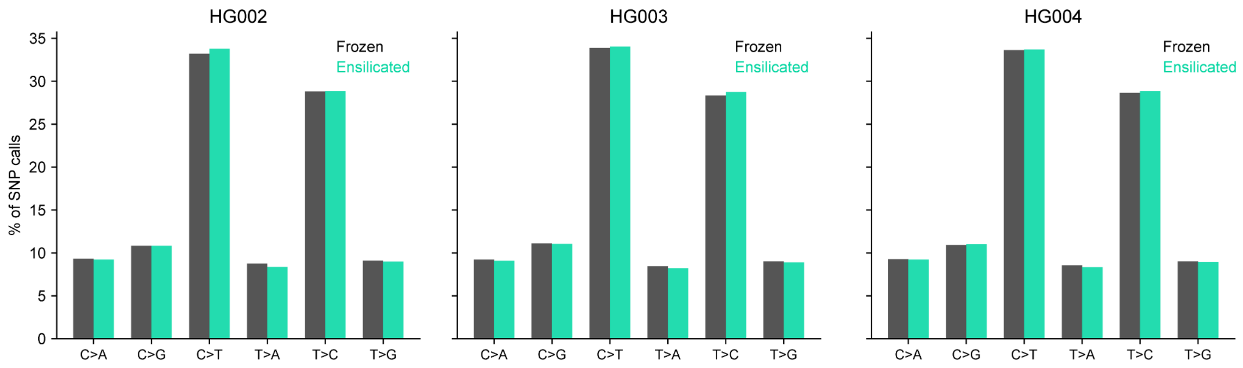

Fig. S4. The relative frequency of each of the six canonical single-nucleotide substitution types is shown for frozen and ensilicated conditions across the GIAB trio samples (HG002, HG003, HG004). Substitution types are reported using the pyrimidine-reference convention: the 12 possible single-base changes are collapsed into six complementary pairs (C>A includes G>T on the opposite strand; C>G includes G>C; C>T includes G>A; T>A includes A>T; T>C includes A>G; T>G includes A>C). Substitution counts were extracted from DeepVariant v1.6.0 whole-genome VCF files (ONT_R104 model) by selecting all biallelic records where both `REF` and `ALT` were single nucleotides (no FILTER restriction was applied; all called SNVs including `PASS`, `LowQual`, and `RefCall` were included, as the goal was to characterize the full mutation spectrum including low-confidence calls where damage artifacts would be most visible). For each VCF, raw counts of each of the 12 REF>ALT substitution types were tallied using awk, then collapsed into the six canonical types by summing complementary pairs. Each bar shows the percentage of all substitutions belonging to that type, computed as the count of that canonical type divided by the total count of all six types × 100. Oxidative DNA damage, e.g., 8-oxoguanine, characteristically elevates C>A transversions; cytosine deamination elevates C>T transitions. Neither signature is enriched in the ensilicated samples relative to frozen, indicating no detectable preservation-induced DNA damage. Input reads were aligned to GRCh38 (GRCh38_GIABv3_no_alt_analysis_set_maskedGRC_decoys_MAP2K3_KMT2C_KCNJ18) with minimap2 v2.28-r1209 (map-ont preset).

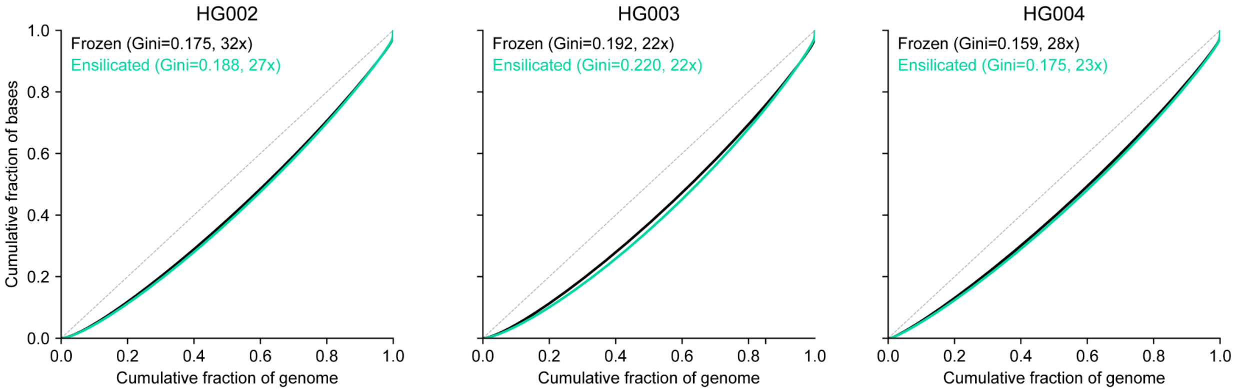

Fig. S5. Lorenz curves showing the cumulative fraction of sequencing bases as a function of the cumulative fraction of the genome, ordered from lowest to highest per-position read depth. A perfectly uniform distribution follows the diagonal. Per-position read depth was extracted from the COV histogram produced by `samtools stats` (v1.21, htslib v1.21), which reports the number of reference bases at each integer coverage depth. Positions were sorted by ascending depth; the x-coordinate at each depth bin is the running cumulative sum of genome fraction (bases at that depth / total bases), and the y-coordinate is the running cumulative sum of depth-weighted bases (depth × bases at that depth / total sequencing yield). The Gini coefficient was computed as $G = 1 - 2A$, where A is the area under the Lorenz curve estimated by the trapezoidal rule. Values near 0 indicate uniform coverage; values near 1 indicate extreme concentration. Mean coverage (shown in legend) was computed as total sequencing yield divided by total reference bases. All reads were aligned to GRCh38 (`GRCh38_GIABv3_no_alt_analysis_set_maskedGRC_decoys_MAP2K3_KMT2C_KCNJ18`) with minimap2 v2.28-r1209 using the `map-ont` preset. No read filtering was applied prior to depth computation.

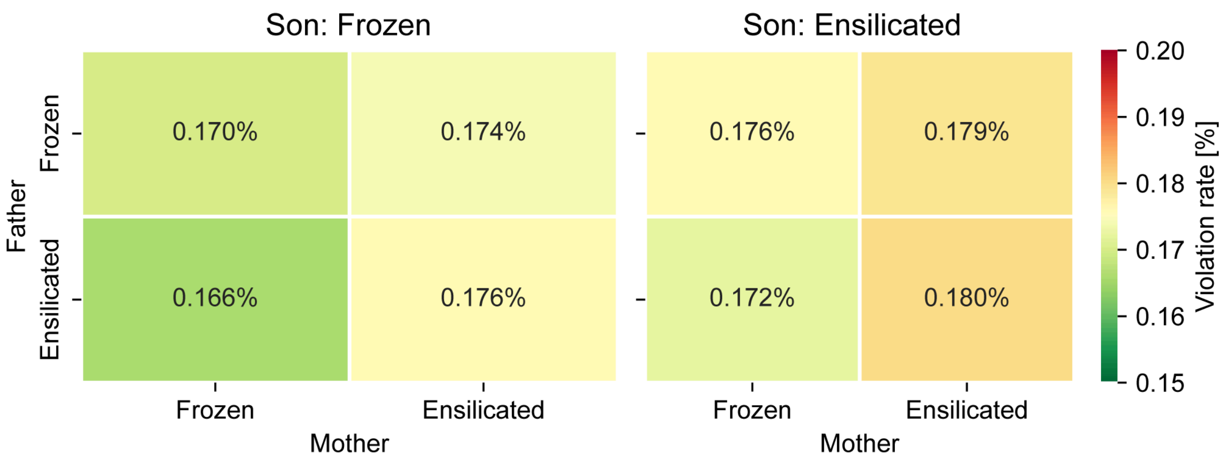

Fig. S6. Heatmaps showing the Mendelian violation rate for all eight combinations of frozen and ensilicated DNA across the GIAB trio (father: HG003, mother: HG004, son: HG002). The left panel fixes the son as frozen; the right panel fixes the son as ensilicated. Within each panel, rows correspond to the father's preservation condition and columns to the mother's. Each cell reports the percentage of sites with a Mendelian-inconsistent genotype in the child given the parental genotypes. Variants were called independently per sample using DeepVariant v1.6.0 with the ONT_R104 model, from reads aligned to GRCh38 (GRCh38_GIABv3_no_alt_analysis_set_maskedGRC_decoys_MAP2K3_KMT2C_KCNJ18) with minimap2 v2.28-r1209 (map-ont preset). Only biallelic single-nucleotide variants (SNVs) passing the FILTER column (PASS or ".") with single-base REF and ALT alleles were retained. For each trio combination, the set of testable sites was defined as positions genotyped in all three individuals. At each site, the child genotype was decomposed into its two alleles: a site was counted as a Mendelian violation if no valid inheritance existed, that is, if no combination of one paternal allele and one maternal allele could produce the observed child genotype. Specifically, for paternal allele set F and maternal allele set M, the child genotype (c_1, c_2) was consistent if any pair (f, m) with $f \in F$ and $m \in M$ satisfied sorted(f, m) = sorted(c_1, c_2). The violation rate was computed as the number of inconsistent sites divided by the total number of testable sites. Approximately 2.3–2.4 million sites were tested per combination, yielding violation rates of 0.166–0.180%, with no systematic difference between preservation conditions.

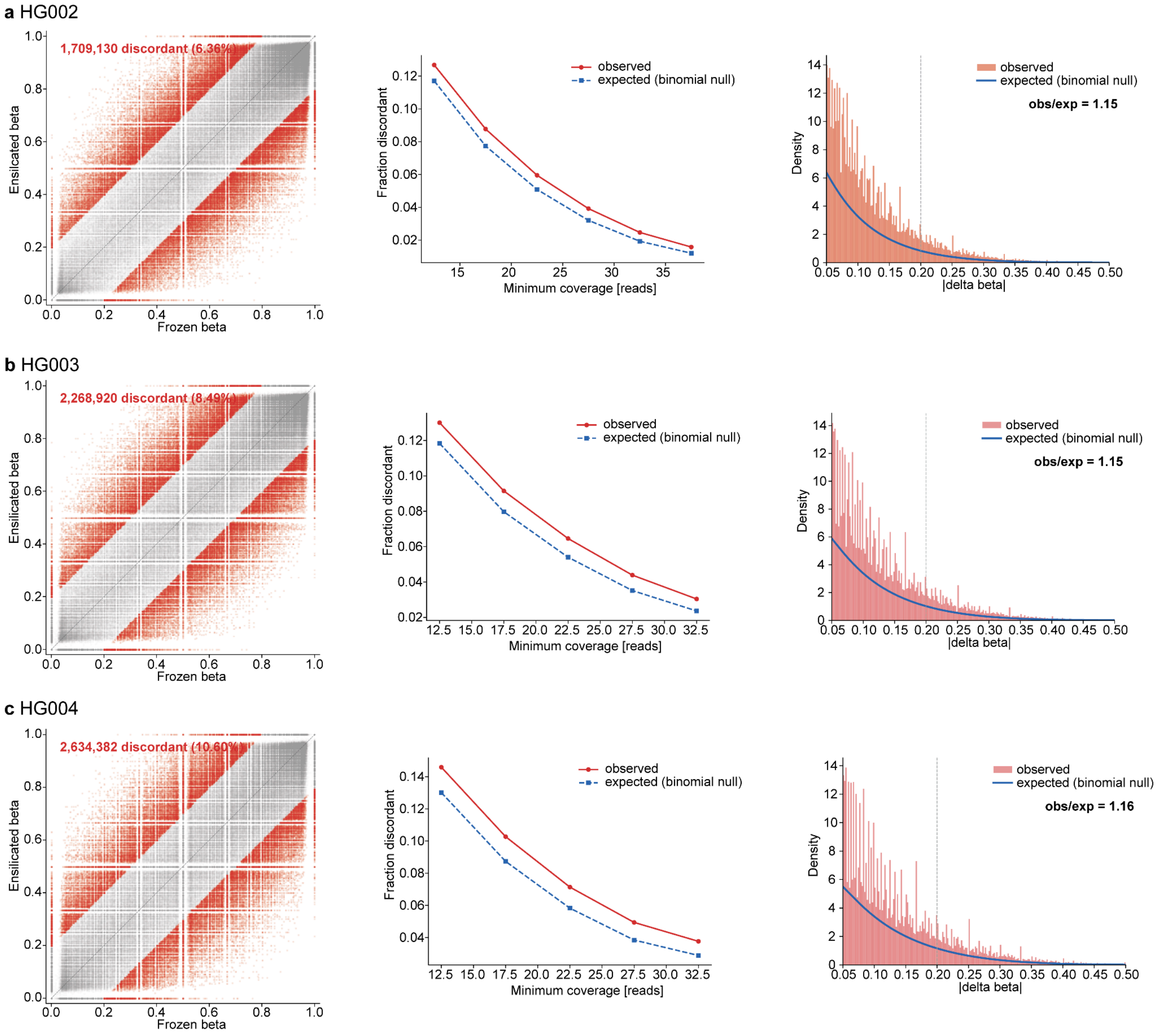

Fig. S7. (Left) Genome-wide methylation scatterplots. For each of the three GIAB trio samples (HG002, HG03, HG004), per-CpG methylation fractions (beta values) from the frozen condition are plotted on the x-axis against the matched ensilicated condition on the y-axis. Methylation was called using `modkit pileup` (v0.3) on minimap2-aligned ONT reads against GRCh38, with the `--combine-strands --cpg --bedgraph` flags, which merges methylation calls from both strands at each CpG dinucleotide and reports the fraction of reads carrying the 5mC modification (beta = modified reads / total reads at that position). Only autosomal and chrX CpG sites with coverage >= 10 reads in both conditions were retained. Concordant sites (|delta beta| <= 0.20) are shown in gray (subsampled to 500,000 for rendering); discordant sites (|delta beta| >0.20) are shown in red (subsampled to 100,000). The dashed line marks y = x. The fraction of discordant sites was 6.36% for HG002, 8.49% for HG003, and 10.60% for HG004, inversely correlated with sequencing depth. (Middle) Observed vs. expected discordance as a function of coverage. At each CpG site, the observed beta values from the frozen and ensilicated conditions were modeled as independent binomial draws from a shared true methylation rate. The pooled estimate of the true methylation fraction was computed as the coverage-weighted average: p-hat = ($beta_{frozen}$ × n_{frozen} + $beta_{ensilicated}$ × $n_{ensilicated}$) / (n_{frozen} + $n_{ensilicated}$), where n_{frozen} and $n_{ensilicated}$ are the read depths at that site. Under the null hypothesis that both samples derive from identical underlying methylation, the

difference ($beta_{frozen}$ - $beta_{ensilicated}$) follows an approximate normal distribution with mean zero and variance p-hat × (1 - p-hat) × ($1/n_{frozen}$ + $1/n_{ensilicated}$). The per-site probability of discordance was then computed as P(|delta| > 0.20) = 2 * [1 - Phi(0.20 / sigma)], where sigma = sqrt(variance) and Phi is the standard normal CDF. Sites were binned by their minimum coverage across the two conditions (5-read bins from 10 to the 99th percentile). Within each bin, the observed discordance rate (fraction of sites with |delta beta| > 0.20) is plotted in red, and the expected rate from the binomial null (mean of per-site discordance probabilities) is plotted in blue. Both curves decline with increasing coverage, confirming that discordance is driven by finite sampling. The observed/expected ratio across all sites was 1.15 for HG002, 1.15 for HG003, and 1.16 for HG004, indicating that the binomial null explains approximately 87% of the observed discordance, with a modest ~15% excess potentially attributable to biological or technical variation. (Right) Distribution of |delta beta| values: observed vs binomial null expectation. The histogram (red, alpha = 0.5) shows the observed distribution of absolute methylation differences across all sites with |delta beta| >= 0.05 (the near-zero peak is excluded for clarity; 225 bins over [0.05, 0.50]). The blue curve shows the expected distribution under the binomial null model, computed as the average folded-normal density across sites. Specifically, for each CpG site with standard deviation $sigma_i$ = sqrt[p-hat_i * (1 - p-hat_i) * ($1/n_{frozen,i}$ + $1/n_{ensilicated,i}$)], the |delta beta| follows a folded normal distribution with density f(x) = 2 * phi(x / $sigma_i$) / $sigma_i$ for x >= 0, where phi is the standard normal PDF. The expected density at each point on the grid (500 points over [0, 0.5]) was computed by averaging these per-site folded-normal densities across a random subsample of up to 2 million sites for computational tractability. The vertical dashed gray line marks the |delta beta| = 0.20 discordance threshold. The close agreement between the observed histogram and the null expectation, particularly in the tail beyond 0.20, confirms that the vast majority of methylation differences are consistent with stochastic sampling from a shared underlying methylation state.

Table S1: Reads were aligned to GRCh38 (GCA_000001405.15, GIAB analysis set with masked GRC decoys and custom MAP2K3/KMT2C/KCNJ18 contigs) using minimap2 v2.28-r1209 with the `-ax map-ont` preset and 5 Gb index batch size (`-K5g`). Alignments were coordinate-sorted with samtools v1.19.2. Mismatch rate was computed by `samtools stats` v1.21 as the number of single-nucleotide mismatches (the mismatches field in the `SN` section) divided by the total number of aligned bases after CIGAR realignment (the bases mapped (cigar) field), expressed as a percentage. This metric counts only substitution errors and excludes insertions and deletions. Supplementary alignments were computed by `samtools flagstat` v1.21 as the number of alignment records with the supplementary flag divided by the total number of primary alignment records (total reads minus secondary alignments), expressed as a percentage. Median per-read identity was computed genome-wide from samtools stats v1.21 for computational efficiency. Samtools stats reports per-read error counts in the RL (readlength) and GCF/GCL sections, and computes a per-read identity from the ratio of matched bases to aligned length across all primary alignments. Specifically, for each alignment record, the aligned length is the sum of all CIGAR-consumed reference and query bases (matches, mismatches, insertions, and deletions), and identity is the fraction of those bases that are sequence matches. The median was taken over all primary, non-supplementary alignments genome-wide, expressed as a percentage.

**Sample**	**Condition**	**Mismatch rate [%]**	**Median identity [%]**	**Supplementary alignments [%]**
HG002	Frozen	2.93	99.05	7.8
	Ensilicated	2.65	99.06	10.8
HG003	Frozen	2.87	99.08	8.0
	Ensilicated	2.60	99.07	12.0
HG004	Frozen	2.85	99.04	12.2
	Ensilicated	2.88	99.00	11.2

Table S2: For each sample and condition, per-read 5mC probabilities were extracted from ONT methylation-tagged BAMs (Dorado v1.4.0, MM/ML auxiliary tags preserved through alignment with `minimap2 -ax map-ont -y`) and compared against an EM-seq consensus truth set. The truth set was constructed from EMseq bedGraph files by retaining only autosomal and chrX CpG sites with coverage >= 10 reads, then classifying sites with EMseq beta >= 0.80 as methylated and beta <= 0.20 as unmethylated; intermediate sites were excluded. For each primary, non-supplementary ONT alignment, the ML tag probability (0-255 unsigned integer) at each CpG was mapped to reference coordinates via `pysam.AlignedSegment.get_reference_positions(full_length=True)` and matched to truth sites. Each single-read call was binarized at a threshold of 128 (p > 0.50 = methylated). Overall accuracy was computed as the fraction of matched observations concordant with the truth label. Sensitivity was computed as the fraction of truth-methylated sites correctly called methylated. Specificity was computed as the fraction of truth-unmethylated sites correctly called unmethylated. All metrics were computed over all matched single-read observations genome-wide (autosomes and chrX).

Sample	Condition	Accuracy [%]	Sensitivity [%]	Specificity [%]
HG002	Frozen	90.26	89.34	93.44
	Ensilicated	90.33	89.42	93.52
HG003	Frozen	86.09	87.99	82.53
	Ensilicated	86.44	88.50	82.53
HG004	Frozen	85.11	83.46	89.74
	Ensilicated	85.49	83.97	89.84

Table S3: Each CpG site with coverage >= 10 reads in both frozen and ensilicated conditions (autosomes and chrX) was annotated relative to CpG islands from the UCSC cpgIslandExt track (GRCh38). For each site, the minimum distance to the nearest CpG island boundary was computed. Sites falling within an island interval were labeled “island”; sites within 0–2 kb of the nearest island boundary were labeled “shore”; sites within 2-4 kb were labeled “shelf”; all remaining sites were labeled “open sea.” Discordant sites were defined as |delta beta| > 0.20 between frozen and ensilicated ONT methylation fractions (`modkit pileup`, `combined-strand`).

Sample	CpG context	% of all sites	Discordant rate [%]
HG002	Island	7.70	1.50
	Shore	7.12	4.08
	Shelf	4.20	4.77
	Open sea	80.98	7.11
HG003	Island	7.70	2.72
	Shore	7.08	5.90
	Shelf	4.15	6.72
	Open sea	81.07	9.35
HG004	Island	8.03	3.42
	Shore	7.35	7.87
	Shelf	4.31	8.96
	Open sea	80.32	11.66

Table S4: For each CpG site passing coverage filters (>= 10 reads in both frozen and ensilicated conditions, autosomes and chrX), local GC content was computed as the fraction of G and C bases in a 500 bp window centered on the site, extracted from the GRCh38 reference using pysam. Discordant sites were defined as |delta beta| > 0.20 between frozen and ensilicated ONT methylation fractions (modkit pileup, combined-strand). Mean GC content and median read depth were computed separately for concordant and discordant site sets. Coverage median was taken over both conditions pooled (frozen and ensilicated read depths concatenated) within each set.

Sample	Discordant [%]	GC concordant	GC discordant	Coverage concordant median [x]	Coverage discordant (median) [x]
HG002	6.36	0.476	0.442	26	23
HG003	8.49	0.476	0.447	22	20
HG004	10.60	0.480	0.454	20	18

Table S5: For each sample, per-CpG beta values from the frozen and ensilicated ONT conditions (modkit pileup, combined-strand) and from EMseq were joined by genomic position (chromosome, start). Sites were restricted to autosomes and chrX and required minimum coverage of 10 reads in all three datasets (frozen ONT, ensilicated ONT, and EMseq). At each site, the absolute difference in beta was computed between each ONT condition and EM-seq ($|beta_{frozen} - beta_{emseq}|$ and $|beta_{ensilicated} - beta_{emseq}|$), and a site was called discordant if this difference exceeded 0.20. Sites were then stratified by the minimum ONT coverage across the frozen and ensilicated conditions at that site, binned into five intervals: 10–15x, 15–20x, 20–25x, 25–30x, and 30x+. Within each bin, the discordance rate was computed as the fraction of sites with |delta beta| > 0.20 relative to EMseq.

**Coverage bin**	**HG002 discordance rate [%]**		**HG003 discordance rate [%]**		**HG004 discordance rate [%]**	
	Frozen	*Ensilicated*	*Frozen*	*Ensilicated*	*Frozen*	*Ensilicated*
10–15x	18.4	20.7	28.1	29.3	29.7	29.7
15–20x	11.6	12.9	27.0	27.5	26.3	26.2
20–25x	9.1	9.7	25.9	26.2	23.6	23.5
25–30x	7.2	7.4	24.1	24.3	21.1	21.1
30x+	5.4	5.6	22.3	22.5	21.3	21.2
